# Animal models of benign airway stenosis: Advances in construction techniques, evaluation systems, and perspectives

**DOI:** 10.1002/ame2.70126

**Published:** 2026-01-23

**Authors:** Wusheng Zhang, Yilin Chen, Chengcheng Yang, Yuchao Dong, Haidong Huang, Hui Shi, Chong Bai

**Affiliations:** ^1^ Department of Respiratory and Critical Care Medicine The First Affiliated Hospital of Naval Medical University Shanghai China

**Keywords:** airway stenosis, animal models, benign airway stenosis, evaluation systems

## Abstract

The incidence of benign airway stenosis (BAS) is on the rise, and current treatment options are associated with a significant risk of restenosis. Therefore, there is an urgent need to explore new and effective prevention and treatment methods. Animal models serve as essential tools for investigating disease mechanisms and assessing novel therapeutic strategies, and the scientific rigor of their construction and validation significantly impacts the reliability of research findings. This paper systematically reviews the research progress and evaluation systems of BAS animal models over the past decade, aiming to provide a robust foundation for the optimized construction of BAS models, intervention studies, and clinical translation. This effort is intended to facilitate the innovation and advancement in BAS prevention and treatment strategies.

## Graphical abstract

Currently, the animals commonly used to establish animal models of benign airway stenosis (BAS) include mice, rats, pigs, dogs, rabbits, and ferrets. The establishment methods involve one or a combination of two methods such as mechanical injury, tracheal cautery, cuff overpressure intubation, laser injury, and endoscopic silver nitrate cauterization. Subsequently, the modeling results are evaluated using methods such as gross and functional evaluation, histopathologic evaluation, imaging assessment, and endoscopic evaluation.

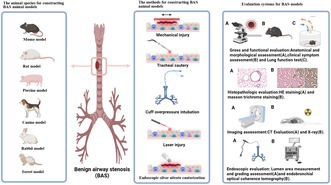

## 1 INTRODUCTION

Benign airway stenosis (BAS) refers to the pathological narrowing of the airway lumen caused by nonneoplastic factors such as iatrogenic injuries, infectious sequelae, or autoimmune diseases. This condition can lead to symptoms such as dyspnea (difficulty breathing), recurrent infections, and respiratory failure, all of which can significantly diminish the patients' quality of life and, in severe cases, may pose a threat to their life.^1^, ^2^, ^3^ As advances in modern medicine, particularly in critical care and respiratory support techniques, continue to progress, the incidence of BAS has been on the rise.^4^, ^5^ Current treatments for BAS primarily include endoluminal interventions (such as balloon dilation and stenting), surgical options, and local drug injections. However, these methods often carry a high risk of restenosis (the renarrowing of the airway), highlighting the urgent need for more effective prevention and treatment strategies.^6^, ^7^, ^8^, ^9^

In recent years, emerging in vitro models—such as organoids, organs‐on‐chips, and human‐derived tissue‐engineered constructs—have greatly advanced research into respiratory diseases.^10^, ^11^, ^12^, ^13^, ^14^, ^15^, ^16^, ^17^, ^18^, ^19^, ^20^ As three‐dimensional tissue analogs cultured in vitro using stem cells, organoids possess the advantages of high humanization and high throughput, and have been widely applied in fields such as disease mechanism elucidation, host‐pathogen interactions, and preliminary drug screening.^10^, ^11^, ^12^, ^13^ By integrating microfluidic and cell engineering technologies, organ‐on‐a‐chip can simulate the complex physiological functions of tissues and organs in vitro.^14^, ^15^, ^16^, ^17^, ^18^ Its highly biomimetic microenvironment and dynamic real‐time monitoring capability enable it to perform excellently in drug permeability and toxicity evaluation, immune‐inflammatory response, and multiorgan toxicity research. Based on engineering principles, human‐derived tissue‐engineered constructs use living cells, biomaterials, and bioactive factors to build living tissue substitutes with specific three‐dimensional structures and physiological functions, showing great potential in personalized disease modeling and regenerative medicine applications.^19^, ^20^ Nevertheless, the aforementioned models still have common limitations: they all lack a complete systemic physiological background (such as systemic immunity, neuroendocrine regulation, and multiorgan coordination) and cannot reproduce the absorption, distribution, metabolism, excretion (ADME) of drugs in living animals, as well as the overall comprehensive efficacy and toxicity. Meanwhile, due to the inherent limitations of human research in terms of ethical regulations and operational feasibility, animal models have become an important tool for basic research.^3^, ^21^, ^22^ They provide controlled experimental platforms for modeling disease processes and understanding mechanisms, as well as for evaluating novel drugs, biomaterials, and medical devices, such as drug‐coated stents.^23^, ^24^, ^25^

How to select an appropriate animal model of BAS is crucial for conducting basic research. This paper systematically analyzes BAS‐related research articles in the past decade and elaborates on the modeling procedures and efficacy evaluation methods, aiming to provide researchers with the basis for model selection, optimize the experimental design, and promote the establishment of standardized BAS animal models, which can accelerate the translation of related basic research results into clinical practice.

## 2 CONSTRUCTION TECHNIQUES OF BAS ANIMAL MODELS

When constructing BAS animal models, a comprehensive consideration of animal anatomical characteristics and research objectives is essential for selecting appropriate injury strategies and technical parameters. This section presents the mainstream modeling methods categorized by animal species (Table 1), with a focus on operational key points and innovative designs (Tables 2 and 3).

**TABLE 1:** Comparative animal models of benign airway stenosis.

Species	Modeling method	Key outcomes	References
Mouse	De‐epithelialization method	Day 14: 66.7% mortality (4/6); luminal narrowing + granulation tissue	[26, 27]
	Mechanical injury method	Day 7: variable stenosis + granulomatous hyperplasia Day 14: tracheal ring fibrosis	
Rat	Mechanical injury method	Day 7: epithelial damage/fibrosis Day 8: lamina propria widening + collagen deposition Day 9: tracheal stenosis: 26.8% ± 4.9%; cricoid stenosis: 48.1% ±2.7% Day 21: 20% mortality; fibrosis in survivors	[28, 29, 30, 31, 32]
Pig	Tracheal cautery (TC)	Day 14: significant stenosis (granulation/inflammation); 33% mortality	[33]
	Cuff overpressure intubation (COI)	Mucosal damage only; no significant stenosis	[33]
	COI‐TC combined group	Day 7: significant stenosis; 12.5% mortality	[33]
Canine	COI	Progressive stenosis: day 7 (7%–18%) → day 14 (14%–53%) → day 21 (21%–64%) + cartilage rupture Days 11–15: 85%–92% stenosis → respiratory distress	[34, 35, 36]
	Stent implantation model	Stenosis: day 7 (9%–10%) → day 14 (15%–18%) → day 21 (36%–47%)	[35]
Rabbit	Mechanical injury method	Time course and severity: Day 7: 60.6% stenosis (95% CI 40.3–80.9) Day 11: significant stenosis Day 21: 59% ± 13% stenosis +30% mortality Day 28: 65.9% stenosis (75% ≥ Myer‐Cotton [MCS] grade II) Week 6–8 month: 100% stenosis rate	[37, 38, 39, 40, 41]
	Mechanical injury method	**Instrument‐dependent effects:** Steel brush: 69.9% stenosis Nylon brush: 15.1% stenosis Polypropylene brush: >86% stenosis (25% mortality) **Injury depth correlation:** Mild mucosal injury: no stenosis Severe mucosal + cartilage injury: 100% stenosis	[42, 43, 44]
	Trachelectomy anastomosis	Day 14: 38.5% ± 2.8% stenosis Day 28: 39.2% ± 4.7% Days 77–82: 27% ± 20% stenosis (grade I)	[45, 46]
	Laser injury method	70 J: 90%–98% stenosis (60% mortality) 50 J: 75%–92% stenosis 40 J: 24%–35% stenosis	[47]
	Tracheal electrocautery combined with tracheal intubation	**50% circumferential cautery + 4‐h intubation:** 80% grade I stenosis (32.7% obstruction) **75% circumferential cautery + 4‐h intubation:** 80% grade II stenosis (62.4% obstruction)	[48]
Ferret	Endoscopic silver nitrate cauterization	100% stenosis: 60% grade II; 40% grade III (MCS)	[49]

Abbreviation: CI, confidence interval.

**TABLE 2:** Comparison of advantages and disadvantages of different modeling methods for benign airway stenosis animal models.

Modeling method	Advantages	Disadvantages	References
Mechanical injury method	Simple operation, low equipment requirements; simulates clinical mechanical injuries (e.g., bronchoscopic procedures, foreign body stimulation)	Difficult to precisely control injury degree; prone to uneven local injury and significant differences in stenosis degree; some animals may develop acute inflammatory reactions due to excessive mucosal injury	[27, 28, 29, 30, 31, 32, 37, 38, 39, 40, 41, 42, 43, 44, 50, 51]
De‐epithelialization method	Specifically removes airway epithelial cells; clear modeling mechanism for stenosis caused by abnormal epithelial injury repair	Delicate and time‐consuming operation; may damage submucosal tissues leading to excessive inflammation or fibrosis; rapid epithelial regeneration in some animals may affect stenosis formation	[26]
Tracheal cautery (TC)	Precise control of cauterization scope and depth; high modeling efficiency; rapid induction of airway tissue necrosis, repair and fibrosis	Excessive cauterization may cause airway perforation and massive hemorrhage; severe local inflammatory reaction; prone to excessive scar tissue hyperplasia after operation	[33]
Cuff overpressure intubation (COI)	Strong clinical relevance (simulates airway injury caused by clinical tracheal intubation cuff compression); relatively simple operation; maintainable injury state	Difficult to control cuff pressure (excessive pressure causes perforation, and insufficient pressure leads to inadequate injury); some animals may have severe cough or laryngeal edema due to intubation stimulation	[33, 34, 35, 36]
COI‐TC combined group	High stenosis formation rate, significant stenosis degree; simulates complex clinical injury scenarios	Tedious operation, severe trauma, strong animal stress response; high complication rate; high requirement for operator proficiency	[33]
Stent implantation model	Simple operation, mild trauma; good animal tolerance, rapid postoperative recovery	Mild injury, low stenosis formation rate (mostly mild stenosis); difficult to form moderate to severe stenosis	[35]
Trachelectomy anastomosis	Precise control of airway defect scope; fixed stenosis location; clear modeling mechanism (simulates stenosis after airway surgical anastomosis)	Severe surgical trauma, complex operation; high requirement for surgical skills; prone to anastomotic leakage, infection or excessive scar hyperplasia after operation	[45, 46]
Laser injury method	Extremely high injury precision (precise positioning and control of injury depth/scope); mild trauma, good hemostatic effect, rapid postoperative recovery	Expensive equipment, high requirement for operating environment; difficult to control laser energy (excessive energy causes perforation, insufficient energy leads to inadequate injury)	[47]
Tracheal electrocautery combined with tracheal intubation	Electrocautery enhances injury degree, intubation maintains injury state; higher stenosis formation rate than single intubation; relatively simpler operation than COI‐TC combined method	Synergistic effect of electrocautery and intubation may cause excessive local injury and increase airway perforation risk; intubation stimulation may aggravate inflammatory reaction at electrocautery site	[48]
Endoscopic silver nitrate cauterization	Precise positioning with endoscope, convenient operation; silver nitrate slowly corrodes airway mucosa to form stable injury; relatively low equipment requirements	Difficult to precisely control silver nitrate corrosion scope (may involve surrounding normal tissues); some animals may have local allergy or strong inflammatory reaction to silver nitrate	[49]

**TABLE 3:** Comparison of critical characteristics of animal modeling for benign airway stenosis.

Modeling method	Key operational points	Mortality rate	Stability	Reproducibility	References
Mechanical injury method	Control injury intensity to avoid airway wall perforation (excessive damage) or failure to induce tracheal stenosis (insufficient damage)	Rabbit: 1/7–4/5 Rat: 1/19–10/10	Moderate (with differences in stenosis degree among individuals)	Good (repeatable after standardized operation, suitable for batch experiments)	[27, 28, 29, 30, 31, 32, 37, 38, 39, 40, 41, 42, 43, 44, 50, 51]
De‐epithelialization method	1. Avoid damaging submucosal blood vessels and cartilage; 2. closely observe airway patency after operation	Mouse:4/6	Moderate (affected by epithelial regeneration rate)	Moderate (requires strict control of de‐epithelialization scope and depth)	[26]
Tracheal cautery (TC)	1. Control cauterization time (usually several seconds) and temperature; 2. stop bleeding in time and prevent infection after operation	Pig:1/3	Good (easy to control stenosis degree, strong model consistency)	Good (repeatable after standardizing cauterization parameters, suitable for studying cicatricial stenosis)	[33]
Cuff overpressure intubation (COI)	1. Maintain a stable cuff pressure (usually 200 mmHg); excessively high or low pressure may affect the stability of the stenosis model; 2. regularly monitor airway patency to avoid tissue necrosis from long‐term compression	Pig:0/3 Canine:2/6–3/3	Moderate (affected by cuff pressure fluctuations)	Moderate (requires strict standardization of intubation operation and pressure control)	[33, 34, 35, 36]
COI‐TC combined group	1. Cauterization scope does not exceed intubation injury area; 2. strengthen postoperative care to prevent complications from double injury	Pig:1/3	Good (stable induction of fibrosis, better stenosis consistency than single method)	Above moderate (requires strict control of parameters and timing of both operations)	[33]
Stent implantation model	1. Select appropriate tracheal tube size; 2. operate gently to avoid damaging larynx and tracheal mucosa; 3. adjust intubation cycle based on experimental needs	‐	Good (all showed mild stenosis)	Good (easy to standardize intubation operation, but repeatability of experimental results affected by mild stenosis)	[35]
Trachelectomy anastomosis	1. Standardized surgical operation process. 2. Strengthen perioperative care	Rabbit: 1/48–1/12；	Good (stenosis degree adjustable by resection length)	Good (precise control of airway defect scope; fixed stenosis location; clear modeling mechanism)	[45, 46]
Laser injury method	1. Select appropriate wavelength laser and adjust energy parameters (usually 8–10 W); 2. avoid damaging surrounding normal tissues	Rabbit: 50 J: 2/7; 70 J: 6/7；40 J: 0/7	Good (consistent stenosis degree, strong model stability)	Good (precisely repeatable after fixing equipment parameters)	[47]
Tracheal electrocautery combined with tracheal intubation	1. Perform electrocautery through intubation gap and control its scope/energy; 2. maintain intubation for a period after operation to enhance injury effect	Rabbit: 0/5	Good (stable induction of moderate‐to‐severe stenosis, better consistency than single method)	Above moderate (requires standardization of electrocautery energy and intubation time)	[48]
Endoscopic silver nitrate cauterization	1. Control silver nitrate dosage and action time; 2. rinse local area with normal saline after operation to reduce continuous injury from residual silver nitrate	Ferret:0/1	Moderate (affected by silver nitrate dosage and action time)	Moderate (easy to standardize endoscopic operation, but repeatability affected by fluctuation of silver nitrate corrosion effect)	[49]

### 2.1 Mouse models

#### 2.1.1 De‐epithelialization method

**FIGURE 1:**
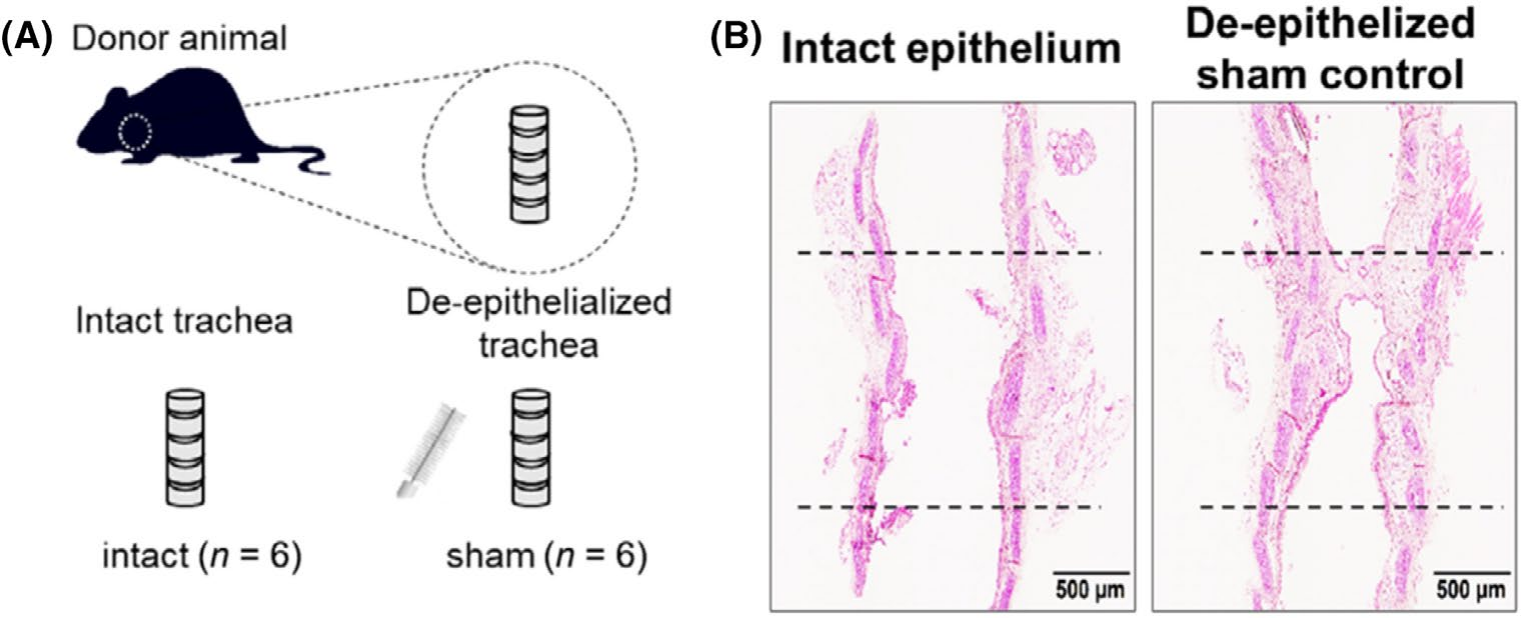
(A) De‐epithelialization method of mouse. (B) Representative hematoxylin and eosin (H&E)‐stained tracheal sections on day 14 after modeling. *Source*: Khalmuratova et al.^26^

Khalmuratova et al.^26^ anesthetized C57BL/6 mice and used a microblade (e.g., no. 11 microblade) to gently scrape the tracheal mucosa along the longitudinal axis, maintaining a shallow angle (15°–30°) to avoid cartilage penetration. Each mucosal area was scraped two to three times until the inner surface appears smooth and lusterless (visual inspection), confirming the removal of the epithelial layer. If physical scraping was suboptimal, a 0.25% trypsin‐soaked cotton swab was applied to the tracheal inner wall for 1–2 min, then the area was rinsed with saline and scraped again to complete epithelial denudation. Assessment performed on day 14 revealed tracheal lumen stenosis with clearly visible granulation tissue hyperplasia (Figure 1A,B).

#### 2.1.2 Mechanical injury method

**FIGURE 2:**
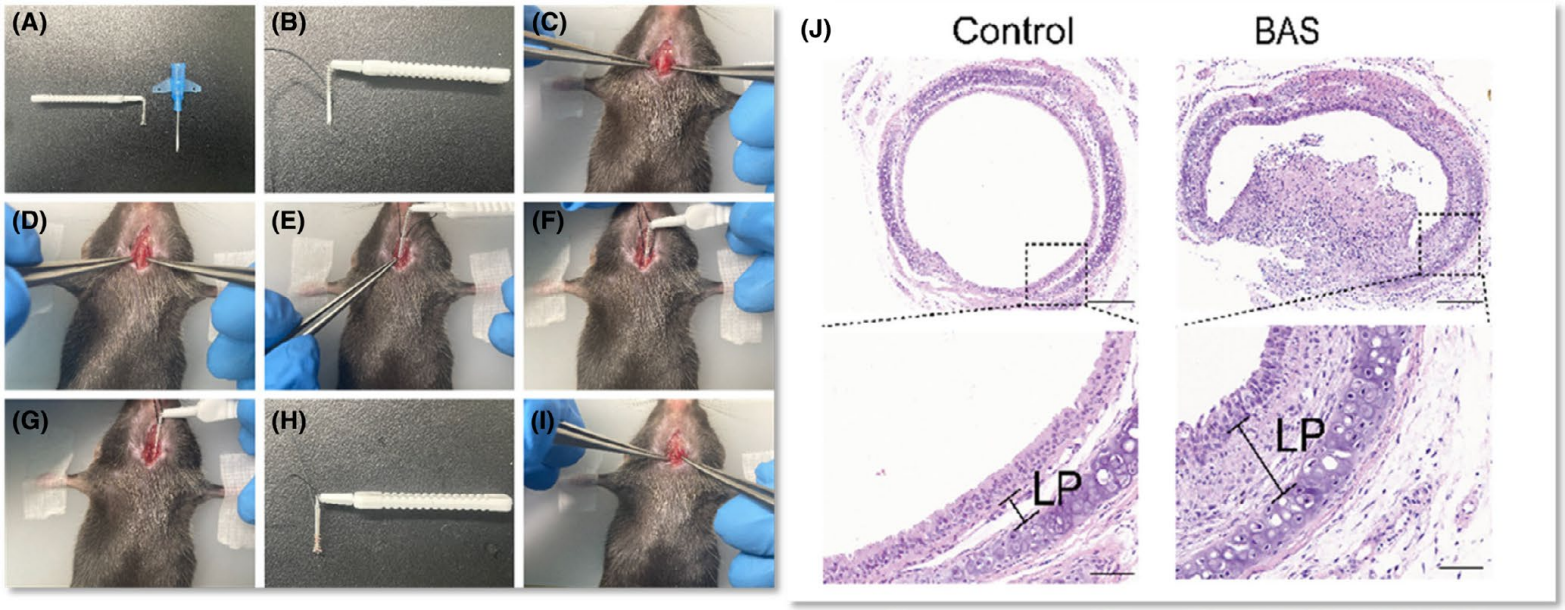
(A–I) Mechanical injury method of mouse. (J) Representative hematoxylin and eosin (H&E)‐stained tracheal sections on day 7 after modeling. Scale bar: 200 μm and 50 μm *Source*: Chen et al.^27^

According to Chen et al.,^27^ after the mice were anesthetized, a transverse tracheal incision was made between two cartilages below the cricoid cartilage, with the incision about two‐thirds of the tracheal circumference. A small brush was then inserted into the trachea through a catheter to a depth of 6 mm. After the catheter was withdrawn, damage was induced by scraping the wall of the trachea with the brush thrice (Figure 2A–I). Fibrosis and airway stenosis were assessed at 7 and 14 days post‐modeling. Seven days after the injury, the tracheas exhibited varying degrees of stenosis, granulomatous hyperplasia, and epithelial cell proliferation. These changes were accompanied by massive inflammatory cell infiltration, excessive and disordered fibroblast proliferation, increased neovascularization, and overdeposition of extracellular matrix. The thickness of the lamina propria (LP) was also notably increased. The results at 14 days indicated the presence of fibrous scarring in the tracheal rings of the mice (Figure 2J).

### 2.2 Rat models

**FIGURE 3:**
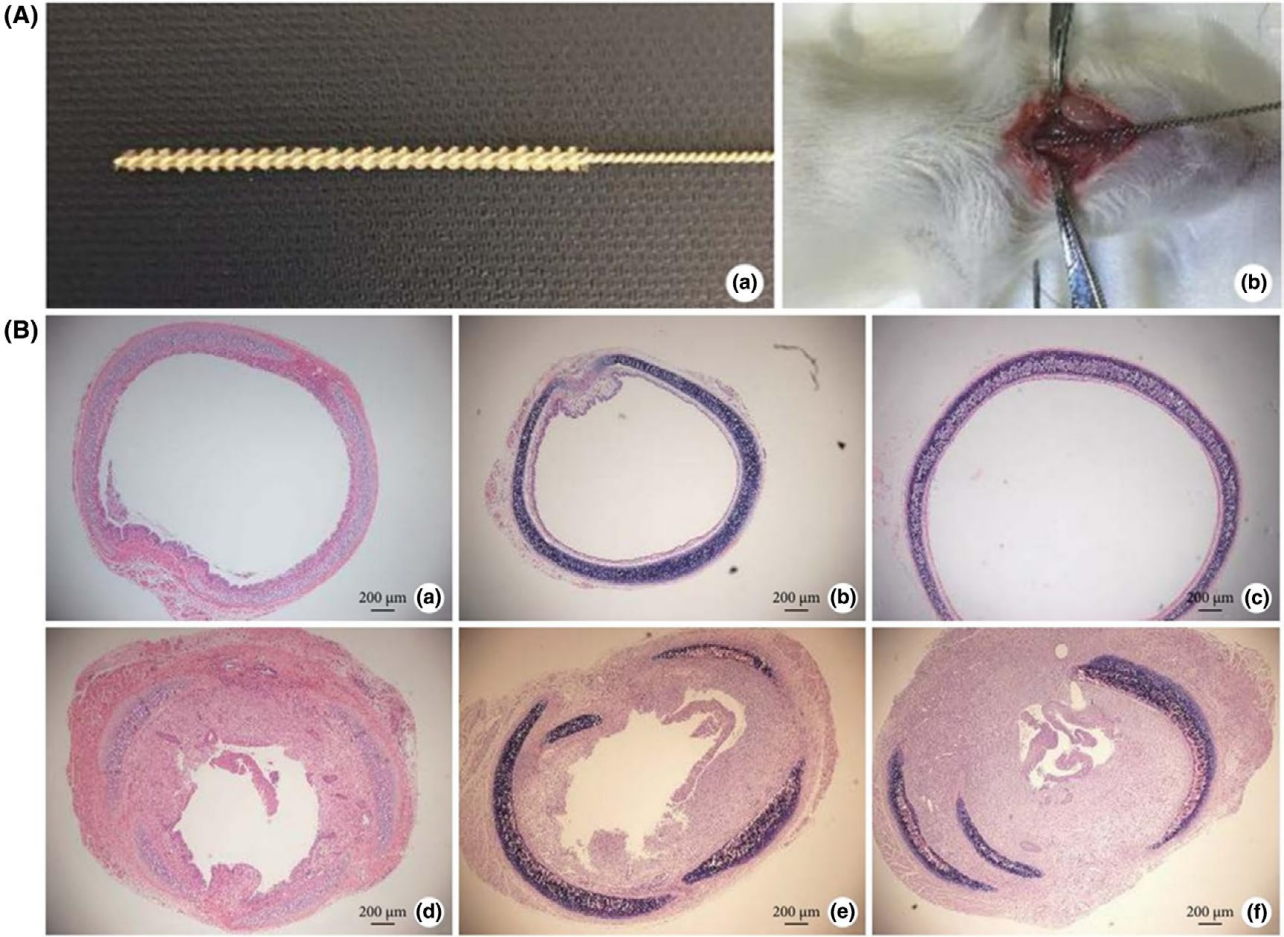
(A) Mechanical injury method of rat. (B) Representative hematoxylin and eosin (H&E)‐stained tracheal sections on day 8 after modeling (a–c: Control group; d–f: Model group). *Source*: Chen et al.^31^

Over the past decade, methods for establishing rat models have predominantly relied on mechanical injury–based approaches.^28^, ^29^, ^30^, ^31^, ^32^, ^50^ Typically, adult Sprague–Dawley (SD) rats are anesthetized, the trachea is surgically exposed, and the mucosal layer is scraped using a nylon brush or micro spatula. Observations were conducted 7–21 days after the procedure, revealing significant airway stenosis. Specifically, Chen et al.^31^ scraped 0.4 cm below the cricoid cartilage and found that the stenosis rate in modeled rats was 60.28% ± 12.56% when assessed on postoperative day 8 (Figure 3A,B). Meanwhile, Mizokami et al.^32^ performed scraping at both the tracheal and cricoid cartilage levels, with mean stenosis percentages of 26.8% ± 4.9% and 48.1% ± 2.7%, respectively.

### 2.3 Porcine models

**FIGURE 4:**
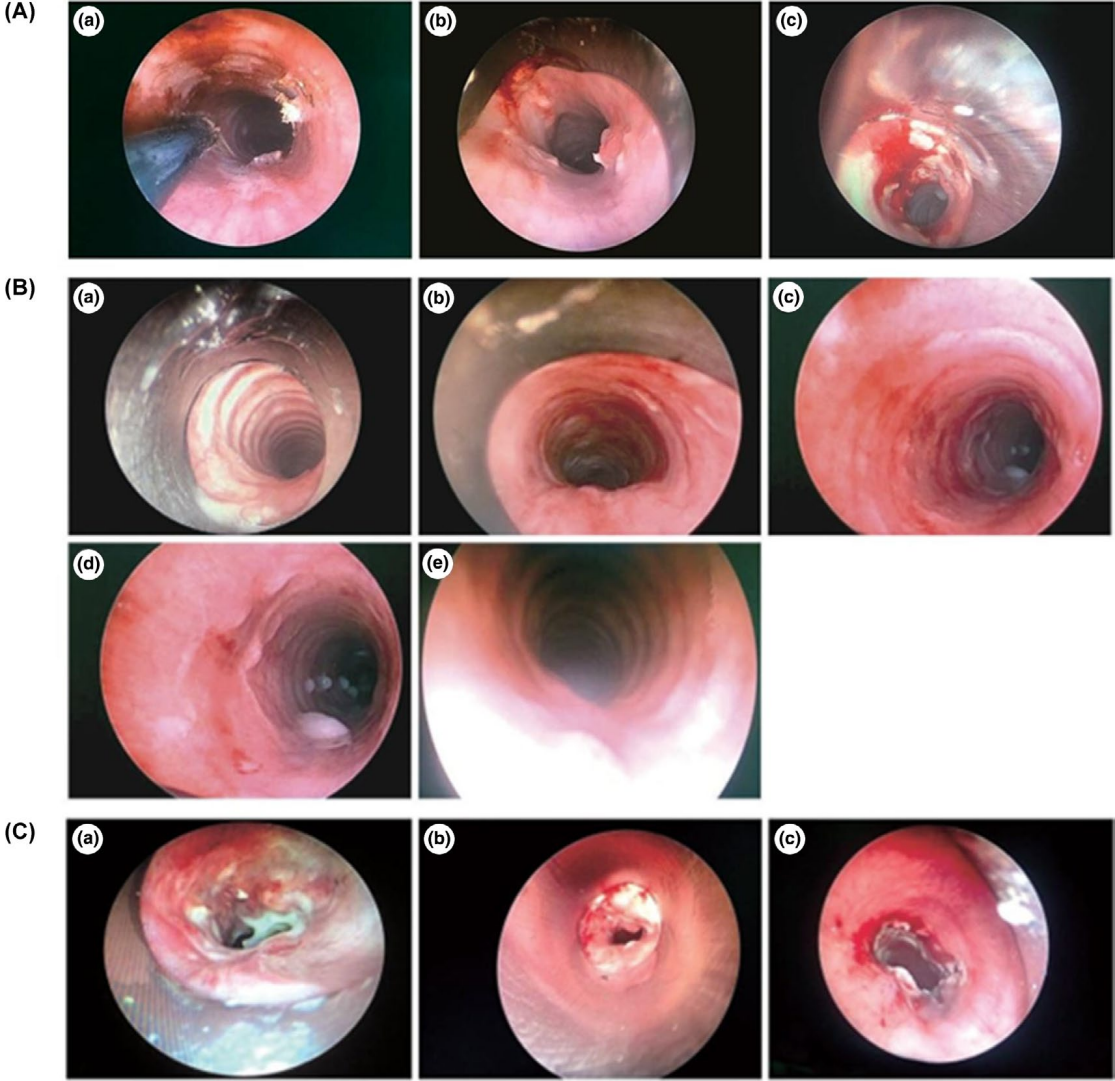
(A): (a) On day 0, 40 W cautery was performed on Pig TC2. (b) On day 7 in Pig TC2, stenosis was not significant (32%). (c) On day 14 in Pig TC2, significant stenosis (65%) had developed as a result of granulation tissue hyperplasia. (B): (a) On day 0 in Pig COI1, before cuff overpressure intubation (COI); (b) on day 0, immediately after 8‐h 200 mmHg COI. (c) On day 0 in Pig COI2, immediately after 8‐h 400 mmHg COI; (d) on day 0 in Pig COI3, immediately after 8‐h 500 mmHg COI; (e): On day 7 in Pig COI3, significant stenosis had not developed (12%). (C): (a) On day 7 in Pig COI‐TC2, significant stenosis (83%) was noted. (b) On day 7 in Pig COI‐TC3, significant stenosis (80%) was noted. (c) On day 7 in Pig COI‐TC7, significant stenosis (50%) was noted. *Source*: Kim et al.^33^

Kim et al.^33^ utilized 12‐week‐old female pigs, selected for their tracheal inner diameter (16–20 mm), which closely resembles that of humans, to establish a model of BAS. The study employed three methods for experimentation and evaluation, which are detailed in Figure 4.

#### 2.3.1 Tracheal cautery

After anesthesia, a rigid bronchoscope (8.5 mm) was used to access the trachea. Cautery was performed using a 10 390 BN cautery suction tube set at 40–60 W. The procedure targeted eight sites, including the membranous portion, within a 1‐cm longitudinal segment located 5 cm below the glottis. The pigs were divided into 60 W and 40 W cautery power subgroups, and a second cautery was performed on day 7. A single cautery resulted in 31%–32% stenosis at 7 days, whereas repeat cautery led to significant stenosis of 63%–65% at 14 days due to granulation tissue hyperplasia. Notably, one pig in the 60 W group experienced sudden death on the seventh day; autopsy revealed a stenosis rate of 34%.

#### 2.3.2 Cuff overpressure intubation

A silicone endotracheal tube (inner diameter 9 mm, outer diameter 12 mm) was inserted, with the cuff positioned 5 cm below the upper incisors to ensure that it was at least 5 cm below the glottis. The cuff was inflated to pressures of 200, 400, or 500 mmHg and maintained for 8 h, with pressure checks and adjustments every 10 min. Despite varying pressures (200–500 mmHg) and intubation durations (4–8 h), no significant stenosis (≤12%) was observed within 2 weeks postintervention. Instead, only mucosal injury, characterized by redness, swelling, and desquamation, was noted, with more severe damage at higher pressures. However, these injuries did not progress to stenosis.

#### 2.3.3 COI‐TC combined group

The combined group was subdivided based on cuff pressure and cuff overpressure intubation (COI) duration as follows: 500 mmHg for 8 h and 4 h; 400 mmHg for 4, 2, 1, and 0.5 h; and 200 mmHg for 1 h and 0.5 h. Tracheal cautery (TC) was performed immediately after COI using 60 W power. Significant stenosis (defined as ≥50% narrowing) was observed within 7 days postoperatively. In the 500 mmHg pressure group, stenosis rates were as follows: on day 7, 89% stenosis was observed in pigs intubated for 8 h, and 83% stenosis was noted in those intubated for 4 h, with an 88% stenosis observed on day 14. In the 400 mmHg pressure group, the stenosis rates for pigs intubated for 0.5–4 h ranged from 53% to 80% on day 7 and increased to 61%–85% on day 14. In the 200 mmHg pressure group, the stenosis rate for pigs intubated for 1 h was 50% on day 7 and 58% on day 14, whereas no significant stenosis was observed in pigs intubated for 0.5 h.

### 2.4 Canine models

**FIGURE 5:**
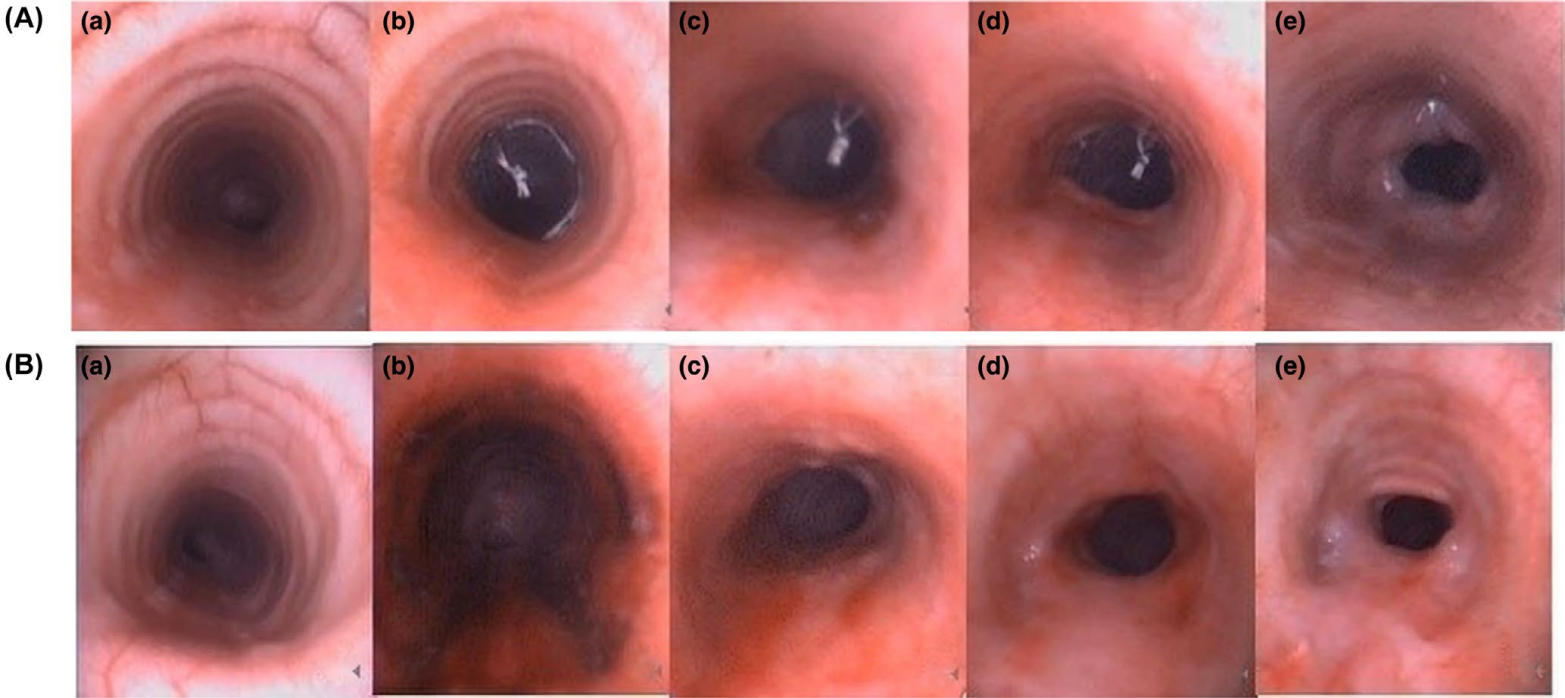
(A): (a) Tracheal morphology before tracheal intubation. (b) Tracheal morphology: 1 day after extubation. (c) Tracheal morphology: 7 days after extubation. (d): Tracheal morphology: 14 days after extubation. (e) Tracheal morphology: 21 days after extubation. (B): (a) Tracheal morphology: Before tracheal stent implantation. (b) Tracheal morphology: 1 day after stent implantation. (c) Tracheal morphology: 7 days after stent implantation. (d) Tracheal morphology: 14 days after stent implantation. (e) Tracheal morphology: 21 days after stent implantation. *Source*: Li et al.^35^

In canine models of BAS, Zhou,^34^ Li,^35^ and Chen^36^ each selected adult Beagles for intervention and used the COI method for modeling. Under laryngoscopic or bronchoscopic guidance, an 8.0–8.5 mm cuffed endotracheal tube was inserted so that the cuff tip was positioned 3–4 cm below the glottis or 28 cm from the incisors. After intubation, cuff pressure was maintained at 200 mmHg for 24 h. Postintervention assessment at 21 days revealed a stenosis rate of 21%–64%^35^ (Figure 5A). Li^35^ also established the model using the stent implantation method. Under the guidance of a bronchoscope, the stent was placed in the trachea, with its distal end positioned 5 cm above the carina to avoid the impact of an excessively high position (prone to stent expulsion or displacement) or an excessively low position (may fall into the bronchus causing obstruction) on the model effect. Postoperative evaluation at 3 weeks showed that a large amount of new granulation tissue formed at both ends of the stent. The surface was covered with yellowish‐white bloody viscous secretions, the tracheal lumen was significantly narrowed, and the degree of stenosis ranged from 36% to 47% (Figure 5B).

### 2.5 Rabbit models

The airway structure of rabbits, such as mucosal layer and cartilage ring layering, is similar to that of humans and easy to manipulate in experiments, which makes it a commonly used animal model in BAS research.^37^, ^38^, ^39^, ^40^, ^41^, ^42^, ^43^, ^44^, ^45^, ^46^, ^47^, ^48^, ^51^

#### 2.5.1 Mechanical injury method

**FIGURE 6:**
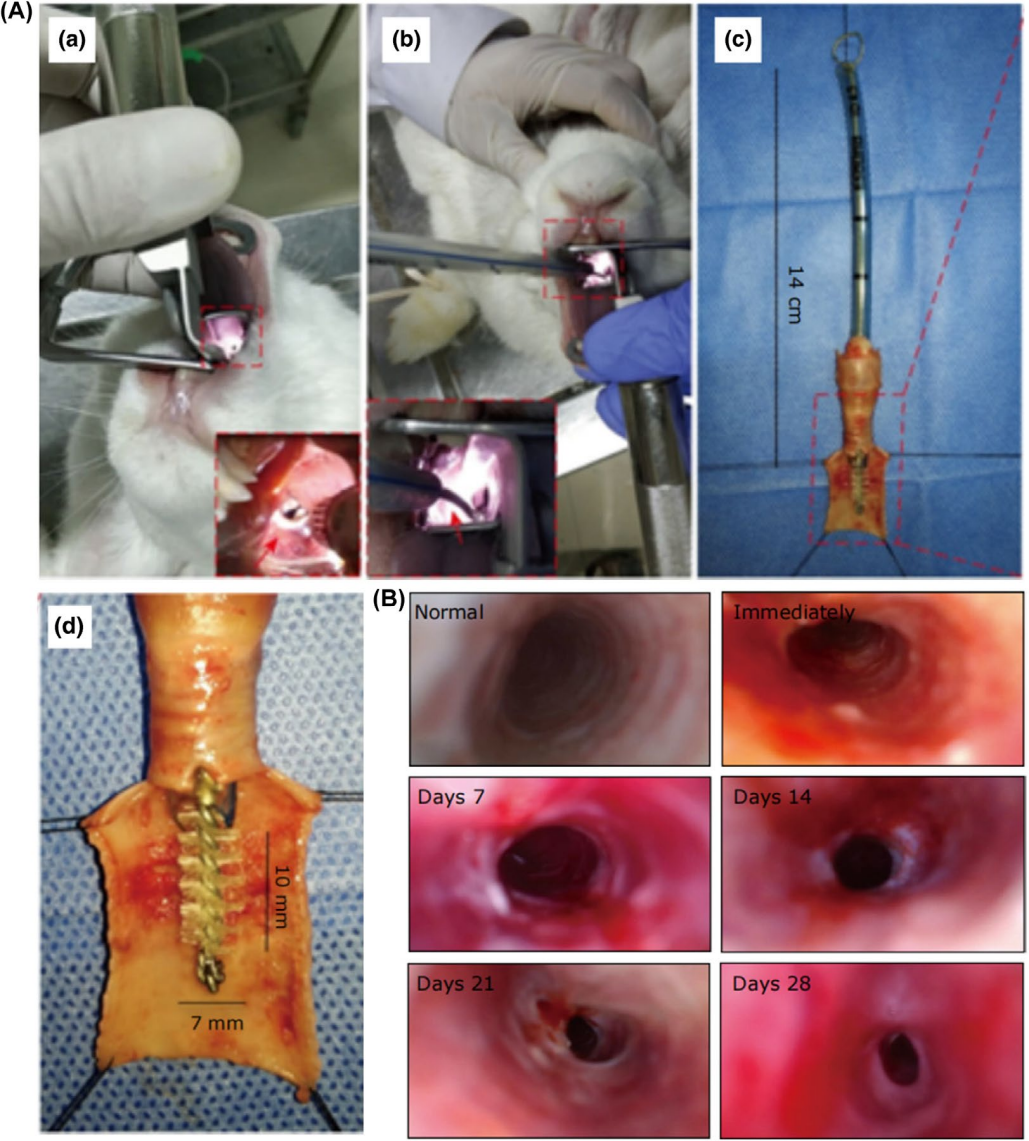
(A) Procedure for establishing a tracheal stenosis rabbit model. (B) Representative images of tracheal cavities in the model group under the handy endoscope. *Source*: Zhang et al.^37^

After anesthesia was induced in adult New Zealand white rabbits, the tracheal lumen was exposed using tracheotomy or oral intubation. A nylon, polypropylene, or steel brush (3.5–7 mm in diameter) was rotated and scraped 10–30 times along the tracheal wall.^37^, ^38^, ^39^, ^40^, ^41^, ^42^, ^43^, ^44^, ^51^ Mild mucosal injury typically resulted in no or minimal stenosis,^41^, ^42^ whereas severe injury involving the cartilage led to significant stenosis (36%–91%, mean ~60%–70%)^37^, ^38^, ^39^, ^43^, ^44^ within 1–4 weeks. In addition, Zhang et al.^42^ successfully induced stenosis by directly disrupting two cartilaginous rings using vascular clamps, with a stenosis rate of 16.59%–76.29% (Figure 6A,B).

#### 2.5.2 Trachelectomy anastomosis

**FIGURE 7:**
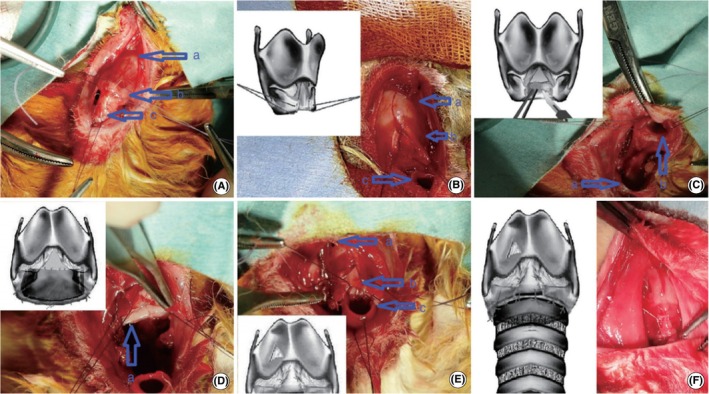
Subglottic stenosis surgery with mucosal graft. (A) Dissection of stenotic trachea segment: (a) thyroid cartilage, (b) cricoid cartilage, (c) stenotik tracheal segment. (B) An incision from the anterior of cricoid cartilage to thyroid cartilage was done, and traction suture was applied to both margins after the stenotic tracheal segment had been removed: (a) thyroid cartilage, (b) cricoid cartilage (anterior part of this is divided, and traction sutures were applied to both margins), (c) distal trachea. (C) The fibrotic mucosa within the cricoid cartilage was excised: (a) distal trachea, (b) cricoid cartilage (leaving the posterior plate of cricoid intact. The tracheal lesion and fibrotic mucosa on the cricoid cartilage were completely removed). (D) The mucosa graft was fixed to cricoid ring: (a) cricoid cartilage (the inner surface of the posterior plate of it was completely covered with mucosal graft). (E) A continuous suture was done between membranous surface of trachea and cricoid ring. Then, the triangle piece obtained from thyroid cartilage was removed, and the cartilage graft was fixed to the anterior surface of cricoid ring: (a) thyroid cartilage (a triangle piece removed from lateral surface of it), (b) cricoid cartilage (the cartilage graft was fixed to cricoid), (c) distal trachea (sutured to the plate of cricoid). (F) Cartilaginous surface of trachea and the anterior surface of cricoid were sutured individually and end‐to‐end anastomosis was completed (the diameter of cricoid ring was enlarged by applying a triangle cartilage graft). *Source*: Aydogmus et al.^46^

Lee et al.^45^ conducted an end‐to‐end anastomosis using 5/0 polydioxanone‐hexacyclone sutures after excising a 2‐cm segment of trachea. At 14 and 28 days postoperatively, except for one case where a rabbit died from tracheal rupture, all surviving rabbits had stenosis (38.5%–39.2%) and fibroblast hyperproliferation. In contrast, Aydogmus et al.^46^ resected second and third tracheal cartilage rings circumferentially, damaged first tracheal ring cartilage and cricoid cartilage inner surface with microscissors, then anastomosed first and fourth tracheal rings end‐to‐end with 5/0 polylactic acid sutures (Vicryl) (Figure 7A–F). The tracheas were evaluated 35–40 days postoperatively using the Myer‐Cotton classification (MCS). MCS is currently the primary international method for assessing subglottic and tracheal stenosis, with the grading criteria as follows: grade I stenosis: the area of luminal obstruction accounts for 0%–50% of the total area; grade II stenosis: the area of luminal obstruction accounts for 51%–70% of the total area; grade III stenosis: the area of luminal obstruction accounts for 71%–99% of the total area; grade IV stenosis: complete luminal occlusion. Results showed that 80% of the rabbits achieved MCS grade I, whereas 20% were classified as MCS grade II. The mean stenosis rate was 27% ± 20%, and there were no deaths in this group.

#### 2.5.3 Laser injury method

**FIGURE 8:**
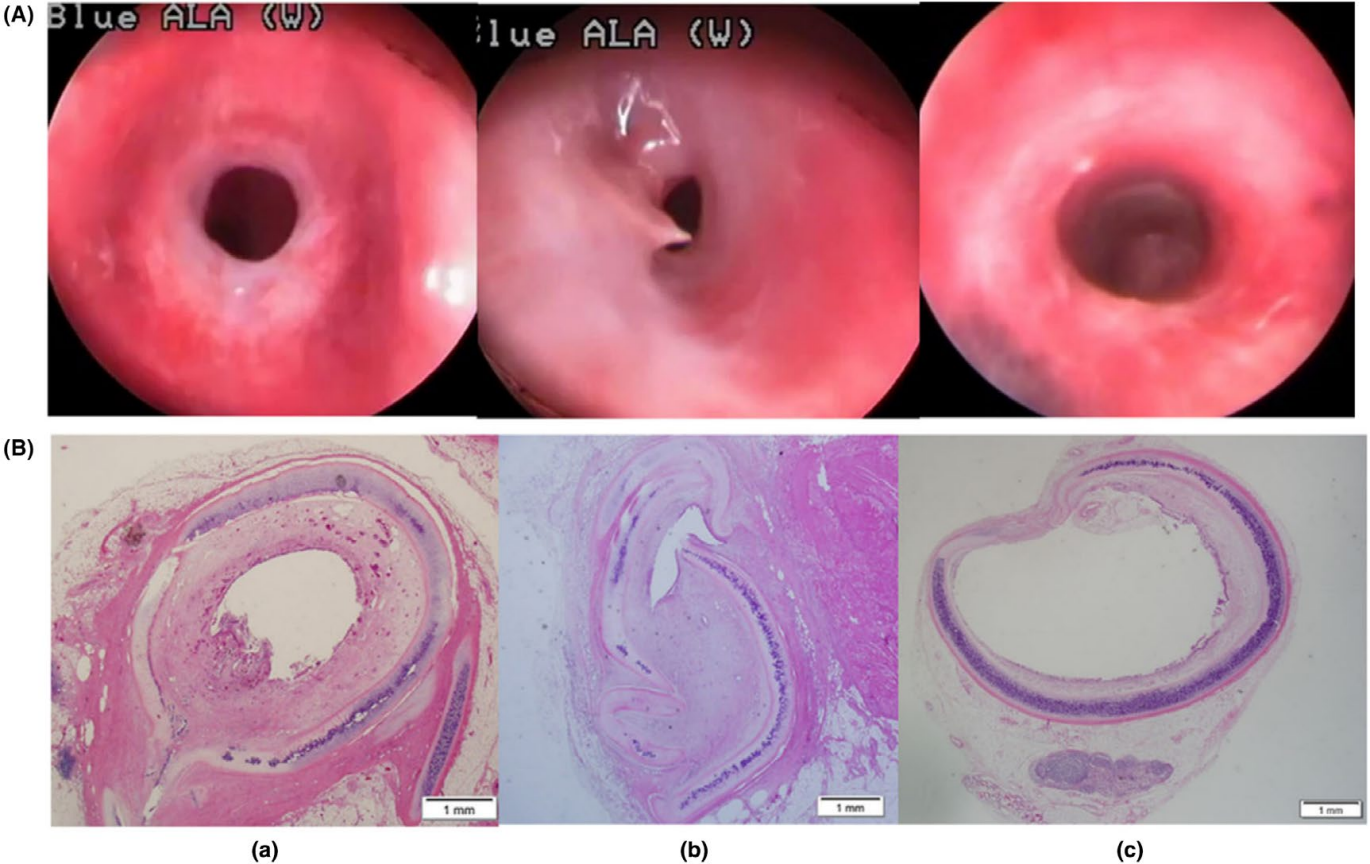
(A) Bronchoscopic view of tracheal stenosis at the time of death in rabbit of group A (84% stenosis at the 28th day) (a), group B (94% stenosis at the 21st day) (b), and group C (28% stenosis at the 28th day) (c). (B) Histologic images of tracheal stenosis at the time of death in group A (77% stenosis) (a), group B (97% stenosis) (b), and group C (25% stenosis) (c). *Source*: Lee et al.^46^

Using bronchoscopic guidance, Lee et al^47^ positioned the diffuser of a diode laser 2–3 cm below the vocal folds in the subglottic region of rabbits for centered irradiation. Rabbits were divided into three groups by laser parameters: group A (10 W, 5 s, 50 J), group B (10 W, 7 s, 70 J), and group C (8 W, 5 s, 40 J). Stenosis rates were monitored continuously for 4 weeks. Results indicated that group A exhibited stenosis rates of 75%–92% (mean 81.1% ± 5.7%), with two animals euthanized at week 3 due to respiratory distress. Group B showed stenosis rates of 90%–98% (mean 94.7% ± 3.3%), with six animals succumbing to severe stenosis or tracheomalacia within 3 weeks and one euthanized on day 16. All animals in group C survived to 4 weeks, with stenosis rates of 24%–35% (mean 28.4% ± 4.1%) (Figure 8A,B).

#### 2.5.4 Tracheal electrocautery combined with COI

**FIGURE 9:**
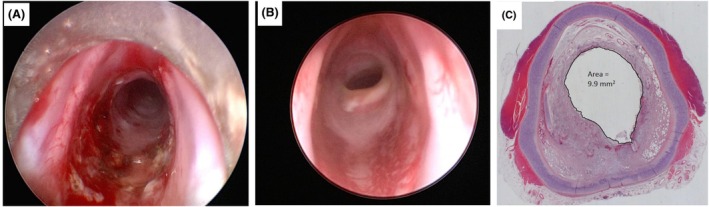
(A) Day 0: Cautery applied to 75% of the circumference of the subglottis. (B) 14 days after injury: Airway endoscopy showing grade 2 subglottic stenosis. (C) 14 days after injury: Hematoxylin and eosin–stained cricoid showing the measurement of its lumen. *Source*: Schweiger et al.^48^

Schweiger et al.^48^ inserted a 3.5‐mm cuffed Portex tracheal tube into the rabbit trachea under direct vision and maintained it for 4 h. Subsequently, 50% or 75% of the posterior subglottic wall circumference was injured using Bugbee monopolar electrocautery (7 W, 5 s). Evaluation at 14 days post‐procedure revealed an obstruction rate of 32.71% in the 50% injury group and 62.44% in the 75% injury group (Figure 9A–C) In another study, Wilson et al.^41^ inserted a 4.0‐gauge cuffed tracheal tube into rabbit trachea and maintained it for 4 h, deflating the cuff every hour. After a 30‐s maneuver involving rapid twisting and proximal and distal movements, the cuff was reinflated and secured. Notably, no tracheal stenosis was observed in these rabbits.

### 2.6 Ferret models

**FIGURE 10:**
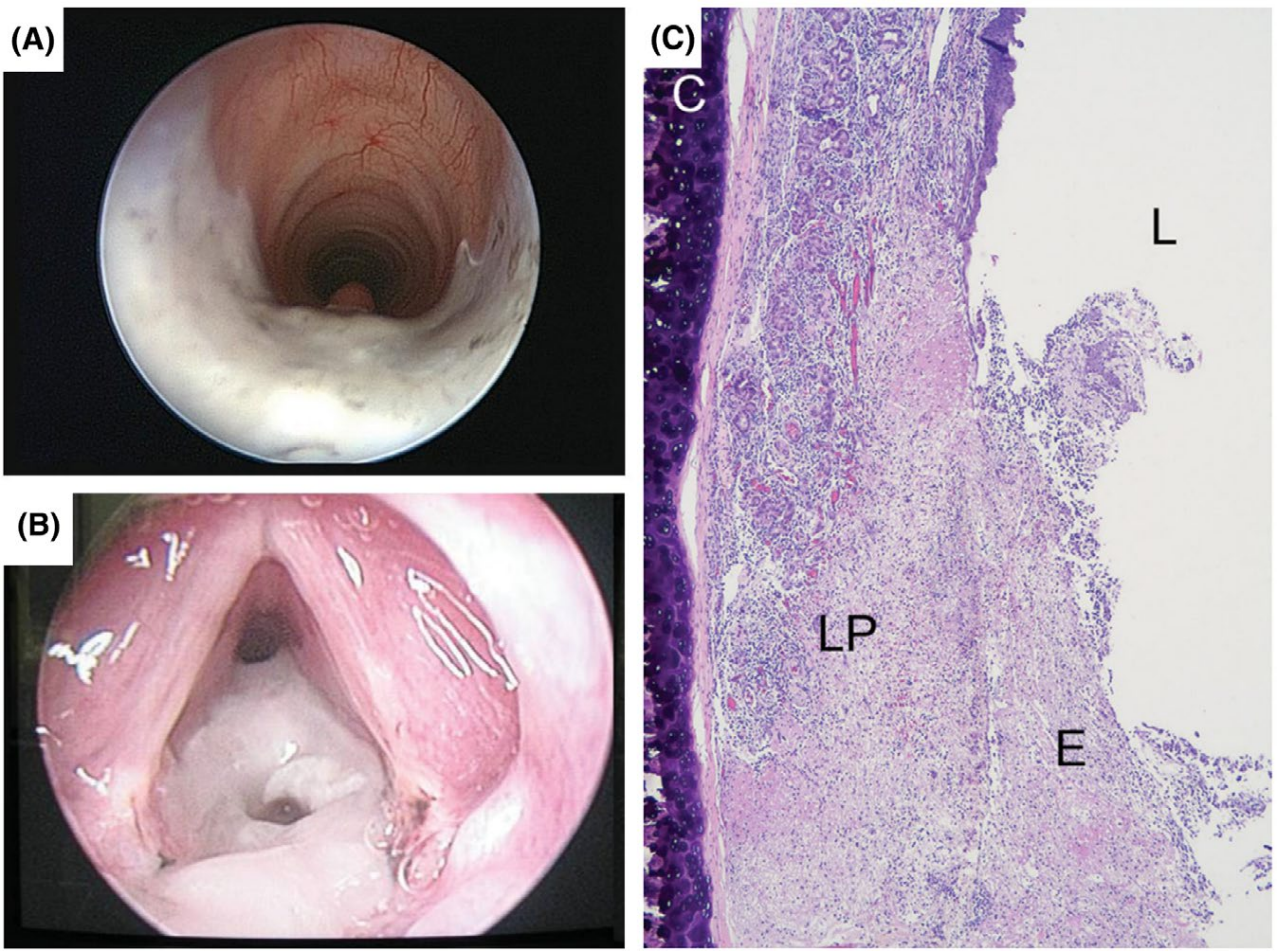
(A) Appearance of ferret subglottis immediately following silver nitrate injury. (B) Ferret larynx/subglottis 3 days after silver nitrate injury. (C) Hematoxylin and eosin slide of a ferret subglottis 3 days after silver nitrate injury to the subglottis (no dilation). Section is at the level of the cricoid cartilage. C, cricoid cartilage; LP, lamina propria/submucosa; E, exudate; L, lumen. *Source*: Tubbs et al.^49^

Tubbs et al.^49^ induced acute subglottic stenosis in 10 adult male ferrets using endoscopic silver nitrate cauterization. After anesthesia, the larynx was exposed using a Miller‐1 laryngoscope, and the airway was observed using a 2.9‐mm Hopkins rod telescope. A dry silver nitrate applicator was then carefully passed through the vocal folds under direct vision and rolled over the posterior‐lateral subglottic mucosa for 5 s to induce subglottic injury. After the applicator was removed, the larynx was gently suctioned to eliminate any residual silver nitrate particles and prevent additional injury. Assessment conducted 48–72 h post‐procedure confirmed the successful induction of acute subglottic stenosis (SGS) in 100% of cases, with stenosis rates of 51%–70% in six cases and 71%–99% in four cases (Figure 10A–C).

## 3 EVALUATION SYSTEMS FOR BAS MODELS

### 3.1 Gross and functional evaluation

#### 3.1.1 Anatomical and morphological assessment

Anatomical and morphological evaluation serves as a critical methodology for investigating the pathological features of tracheal stenosis. Direct anatomical observation combined with imaging methods allows for a systematic analysis of the particular tracheal stenosis condition.^42^ In a study conducted by Aydogmus et al.,^46^ tracheal specimens were separated, and the inner and outer diameters of the cartilage rings were precisely measured using microscopic measurement instruments. This made it possible to build a model of the narrowed region's spatial localization. The MCS criteria were then used to standardize and evaluate the tracheal narrowing (Table 4): grade I stands for lumen blockage <50%, grade II for obstruction between 50% and 70%, grade III for obstruction between 70% and 99%, and grade IV for full lumen obstruction. This quantitative grading approach facilitates an intuitive and accurate assessment of the severity of tracheal narrowing.

**TABLE 4:** Formula for calculating the degree of stenosis.

Calculation formula	Explanation of formula parameters	Application scenarios	References
Stenosis rate (%) = (1 − *s*/*S*) × 100%	*s*: luminal area of the most stenotic part of the airway after injury treatment; *S*: nearest normal area: the beginning of the stenosis or 1 cm/3 mm above the nearest normal tracheal ring	Dynamic observation of airway stenosis in modeled animals during the modeling cycle (e.g., weeks 1, 2, 3, and 4), or assessment of tracheal stenosis severity at the experimental endpoint can be performed using CT, bronchoscopy, and other techniques. Additionally, MCS can be employed to grade the severity of stenosis and evaluate the success of modeling	[27, 37]
Stenosis rate (%) = (1 − *s*/*S*) × 100%	*s*: the area of the most stenotic segment of the lumen after modeling; *S*: the original area of the stenotic segment before modeling		[33, 35, 38, 44, 47]
Stenosis rate (%) = (1 − *s*/*S*) × 100%	*s*: luminal area of the most stenotic part of the airway after injury treatment; *S*: luminal area of the corresponding part in the normal uninjured airways of the control group		[48]
Stenosis rate (%) = (1 − *s*/*S*) × 100%	*s*: area of the most severely stenotic segment, calculated as *s* = *π*[(*d*_1_ + *d*_2_)/2],^2^ where *d*_1_ represents the longest diameter of the ventilation lumen, and *d*_2_ represents the shortest diameter; *S*: annular area enclosed by the cartilage ring, calculated as *S* = *π*[(*D*_1_ + *D*_2_)/2]^2^, where *D*_1_ denotes the longest diameter of the cartilage ring, and *D*_2_ denotes the shortest diameter	The method proposed by Nakagishi is used for the dynamic observation during the modeling cycle and the assessment of the overall degree of stenosis at the experimental endpoint.	[31, 32, 43]
Stenosis rate = [1 − (*d*_1_ × *d*_2_)/(*D*_1_ × *D*_2_)] × 100%	*d*_1_ and *d*_2_: the transverse and longitudinal diameters of the narrowest lumen; *D*_1_ and *D*_2_: the transverse and longitudinal diameters at 1 cm below the narrowest segment or at the level of the cartilage ring	Dynamic observation during the modeling cycle and the experimental observation endpoint	[39, 42]
Percentages of stenosis = (1 − *d*/*D*) × 100%	The average values of the outer boundary diameter (*D*) and inner boundary diameter (*d*) of the cricoid cartilage along two axes	Modified MCS for the experimental observation endpoint	[46]
Stenosis index = [1 − (*d*_1_ + *d*_2_)/(*D*_1_ + *D*_2_)] × 100%	*d*_1_ and *d*_2_: the anteroposterior diameter and transverse diameter of the stenotic tracheal segment; *D*_1_ and *D*_2_: the anteroposterior diameter and transverse diameter of the normal tracheal segment	Experimental observation endpoint	[50]

Abbreviations: CT, computed tomography; MCS, Myer‐Cotton classification.

#### 3.1.2 Clinical symptom assessment

Researchers routinely evaluate airway obstruction‐related signs and symptoms using several criteria: (1) respiratory symptoms: These include characteristic wheezing, the presence of triad signs, and an abnormal respiratory rate; (2) systemic manifestations: indicators such as decreased exercise tolerance and weight loss due to feeding difficulties are considered; (3) critical signs: this category includes respiratory distress, cyanosis, and other acute manifestations of hypoxia.^30^, ^36^, ^39^, ^41^, ^48^ These symptoms directly reflect the degree of physiological compromise caused by airway narrowing. Thus, they provide essential clinical validation for determining model efficacy.

#### 3.1.3 Lung function test

In the evaluation of BAS in animal models, noninvasive pulmonary function testing (PFT) provides a critical tool for quantifying pathophysiological abnormalities.^27^ Key parameters, such as prolonged inspiratory phase duration, serve as indirect indicators for increased airway resistance, bronchoconstriction, or luminal narrowing. This longitudinal monitoring strategy enables real‐time assessment of airway patency and disease progression while adhering to established animal welfare standards.

### 3.2 Histopathological evaluation

#### 3.2.1 HE staining

Hematoxylin and eosin (H&E) staining enables systematic analysis of multifaceted histological alterations in tracheal stenosis models. This methodology facilitates the evaluation of mucosal epithelial integrity through assessment of architectural continuity and ulceration; characterization of inflammatory cell infiltrates, including lymphocyte, neutrophil, and eosinophil populations; quantification of granulation tissue proliferation via neovascularization density and fibroblast activation status; and histopathological examination of cartilaginous destruction patterns such as matrix degradation and calcified deposit formation.^26^, ^27^, ^28^, ^29^, ^30^, ^31^, ^32^, ^33^, ^34^, ^35^, ^36^, ^37^, ^42^, ^50^, ^51^ These histological parameters collectively elucidate pathological progression mechanisms underlying airway stenosis, providing critical insights into disease pathogenesis and therapeutic response evaluation.

#### 3.2.2 Masson trichrome staining

Masson trichrome staining serves dual analytical purposes in tracheal stenosis research. First, it enables quantitative assessment of collagen deposition through the calculation of the collagen volume fraction (CVF), providing precise measurement of tissue fibrosis severity. Second, this technique facilitates spatial distribution analysis by clearly delineating fibrotic differences between the submucosal layer and cartilaginous matrix.^27^, ^28^, ^29^, ^30^, ^31^, ^37^, ^39^, ^43^, ^50^, ^51^ Such dual‐modality evaluation enhances the understanding of layer‐specific fibrotic progression during airway narrowing, offering critical insights into pathological remodeling mechanisms.

#### 3.2.3 Detection of fibrosis‐related molecular proteins

Immunohistochemistry (IHC) and Western blotting (WB) can be used to detect the protein expression levels of myofibroblast activation markers such as α‐smooth muscle actin (α‐SMA) and fibroblast activation protein (FAP), as well as extracellular matrix components, including type I collagen (COL1) and type III collagen (COL3).^27^, ^28^, ^30^, ^36^, ^40^, ^50^, ^51^ Immunofluorescence (IF) allows for in situ localization and semiquantitative analysis of specific cells and proteins; for instance, total macrophages can be labeled via CD68, or profibrotic M2‐type macrophages can be identified using CD163 and Arg1.^26^, ^27^, ^28^, ^50^

Based on the aforementioned pathological staining results, quantitative analysis can be performed using image processing software such as ImageJ and QuPath or artificial intelligence segmentation technologies such as U‐Net.^26^, ^50^, ^51^, ^52^, ^53^, ^54^, ^55^ This strategy, which combines conventional staining, specific markers, and quantitative tools, enables the analysis of lesion characteristics from morphological, molecular, and statistical multidimensions. Using multiparameter cross‐validation, this evaluation system ensures the objectivity and accuracy of the results, laying a solid pathological foundation for model validation and therapeutic efficacy assessment.

### 3.3 Imaging assessment

Imaging assessment plays an important role in evaluating BAS animal models due to its noninvasive advantages, which can monitor the structural changes in the airway in real time.

#### 3.3.1 CT evaluation

High‐resolution computed tomography (HRCT), micro‐CT, and conventional CT techniques enable precise measurement of tracheal inner diameter and calculation of stenosis rate.^27^, ^35^, ^42^ Among these techniques, Micro‐CT has made tremendous contributions to respiratory disease research in recent years, owing to its submillimeter resolution that allows clear visualization of tracheal microstructural changes.^56^, ^57^, ^58^ For instance, Mohamady,^56^ Schwab,^57^ and Vermaut^58^ et al. used micro‐CT to investigate small airway lesions in chronic obstructive pulmonary disease, pneumonia, and the status of terminal pulmonary arterioles and small airways in advanced cystic fibrosis. Similarly, Chen et al.^27^ applied micro‐CT to obtain submillimeter‐resolution imaging of the mouse trachea, facilitating detailed visualization of microstructural alterations.

#### 3.3.2 Optical coherence tomography

Optical coherence tomography (OCT) is a noninvasive (or minimally invasive) imaging technique based on the principle of low‐coherence interferometry, enabling high‐resolution cross‐sectional imaging of biological tissues with a resolution reaching the micrometer level—far superior to that of traditional ultrasound and CT technologies. It can clearly distinguish the boundaries of the airway's mucosal layer, submucosal layer, and cartilage layer (if penetration is sufficient) and accurately measure the thickness of each layer. Prior to the formation of macroscopically visible stenosis caused by fibrosis, it can detect early thickening and structural disorganization of the submucosal layer. Furthermore, semiquantitative assessment of collagen deposition and scar tissue formation can be achieved by analyzing the backscattering characteristics of OCT signals (e.g., changes in grayscale values).^34^, ^42^, ^59^, ^60^ For instance, Xu et al.^60^ used it to evaluate airway wall compliance during inhalation injury responses, whereas Zhang^42^ and Zhou^34^ employed OCT for dynamic assessment of the progression of airway stenosis.

#### 3.3.3 Fluorine‐18‐FAP inhibitor positron emission tomography/CT

Fluorine‐18‐FAP inhibitor positron emission tomography/CT (18F‐FAPI PET/CT) is an advanced molecular imaging technique that targets FAP to visualize activated fibroblasts in living tissues. It can not only assess the degree of fibroblast activation by detecting FAP uptake but also calculate the stenosis index by measuring tracheal diameter.^51^, ^61^ Luo et al.^51^ used it to dynamically assess the degree of fibroblast activation during the progression of BAS and calculate the stenosis index by measuring the tracheal diameter.

#### 3.3.4 X‐ray

In the evaluation of BAS animal models, X‐ray examination is mainly used for measuring the inner diameter of the anastomosis. The stenosis rate is calculated by the ratio of the minimum lumen area to the normal area.^45^ It is relatively simple to operate and can provide a certain reference basis for the assessment of airway stenosis.

In studies on animal models of BAS, various imaging techniques exhibit distinct characteristics: X‐ray, featuring low cost and ease of operation, is suitable for preliminary screening and assessment of complications but fails to directly visualize airway details. Conventional CT and HRCT can clearly display the three‐dimensional anatomical structure of the airway and the degree of stenosis, with the latter offering higher resolution and serving as the preferred choice for anatomical evaluation; however, neither can effectively distinguish the activity of fibrosis. Micro‐CT, with extremely high resolution, is applicable for terminal ex vivo verification but cannot perform dynamic in vivo monitoring. OCT enables “optical biopsy” of the airway mucosa, making it suitable for observing early superficial lesions, yet its penetration depth is limited. 18F‐FAPI PET/CT, by targeting fibroblast activation protein, noninvasively and dynamically reflects fibrotic activity at the molecular level, and is particularly suitable for efficacy evaluation. In practical applications, the synergy of multiple techniques can comprehensively reveal the disease process: HRCT is used for long‐term noninvasive follow‐up of anatomical changes, OCT for detecting early submucosal lesions, and 18F‐FAPI PET/CT for revealing the underlying molecular activity, with final high‐precision verification via micro‐CT or histology, thereby comprehensively and deeply elucidating the disease process.

### 3.4 Endoscopic evaluation

Bronchoscopy is the core tool for evaluating the modeling effect. It not only can directly observe airway lumen stenosis, granulomatous hyperplasia, and mucosal lesions in living animals, but also dynamically assess each process of stenosis occurrence through endobronchial OCT (EB‐OCT).^26^, ^33^, ^34^, ^38^, ^41^, ^44^, ^47^

The macroscopic and functional assessments provide a holistic overview of the model from the macroscopic level, elucidating how airway stenosis impacts animal physiological functions. Histopathological assessments penetrate the cellular and tissue levels to analyze the nature of the lesions and the pathological evolution mechanism. Imaging assessments, with the advantage of noninvasiveness, monitored the changes in the airway structure in real time. The endoscopic assessments were able to observe the internal condition of the airway directly in vivo to judge the modeling effect accurately. Endoscopic evaluation can directly observe the internal condition of the airway in vivo and precisely determine the effect of modeling. Each method has its advantages, but they may have limitations when used alone. Therefore, a three‐dimensional assessment framework of “morphology‐function‐pathology” can be established when constructing an animal model of BAS. The combination of imaging and endoscopy can provide accurate anatomical quantification to guide the selection of biopsy sites; functional monitoring can help establish the correlation between clinical symptoms and objective indicators; histopathological verification can confirm the nature of the lesion and effectively differentiate between reversible inflammation and irreversible fibrosis; and standardized postoperative dynamic follow‐up protocols can be formulated to capture the changes in the model promptly. Through multiparameter cross‐validation, this assessment framework enables systematic and comprehensive evaluation of stenosis modeling efficacy, providing a robust methodological foundation for subsequent intervention studies.

## 4 CHARACTERISTICS AND APPLICATION SCENARIOS OF MAJOR BAS ANIMAL MODELS

Animal models are essential for studying the pathological mechanisms of biological systems and for evaluating the safety and efficacy of new therapies, including drugs, devices, and biomaterials. When selecting a suitable model, it's important to consider factors such as anatomical and physiological similarities, cost, maneuverability, ethical considerations, and research objectives.

### 4.1 Rodent models (mice, rats)

**Advantages:** (1) *Cost efficiency*: The purchase, feeding, and experimental operational costs for small animals are significantly lower compared to large animal models, offering superior cost‐effectiveness.^62^, ^63^, ^64^ (2) *Rapid reproduction and transparent genetic background*: Short‐generation times enable the rapid production of genetically homogeneous cohorts (including inbred strains) or genetically modified models (e.g., transgenic, knockout). This facilitates the targeted investigation of specific gene roles in stenosis pathogenesis (e.g., fibrosis, inflammation).^64^, ^65^, ^66^ (3) *Immunological tool availability*: A wide variety of antibodies and molecular tools are available, enabling in‐depth investigation of immune responses and inflammatory mechanisms.^1^, ^27^

**Limitations:** (1) *Differences in airway structure*: The airway structure in mice is significantly different from that of humans. For instance, the diameter of the trachea is much smaller, the cartilage rings are closed rather than open, the distribution of submucosal glands is minimal, and the mechanism of the cough reflex differs greatly from that in humans. These anatomical differences restrict the use of mice for modeling large airway stenosis in humans, evaluating intraluminal devices (such as stents), or studying physiological clearance mechanisms like coughing.^67^, ^68^, ^69^ (2) *Variations in disease progression*: Rodent models may exhibit accelerated stenosis formation/resolution kinetics compared to humans, with potential absence of complex chronic inflammatory and remodeling processes characteristic of human disease.^70^, ^71^, ^72^

Therefore, rodent models are more suitable for: (1) *mechanistic studies*: These studies aim to explore the roles of various biological responses, including inflammation (such as macrophage and neutrophil polarization), fibrotic pathways (like transforming growth factor beta [TGF‐β] and epithelial‐mesenchymal transition), neovascularization, and epithelial repair in the formation of stenosis. (2) *Early‐stage drug screening and mechanism validation*: Rodents can be used to quickly assess the preliminary efficacy of various treatments, including anti‐inflammatory, antifibrotic, and antiproliferative drugs, as well as innovative biologics (such as antibodies and gene therapies). This helps in evaluating both the efficacy and mechanisms of action for new therapeutic options.

### 4.2 Large animal models (e.g., pigs, dogs)

**Advantages:** (1) *Similarity in airway structure*: The size and structure of the airway in these models closely resemble those of humans. These models exhibit significant anatomical fidelity to human airways, encompassing tracheal/bronchial dimensional equivalence, C‐shaped cartilage morphology, submucosal gland distribution, and ciliary motility dynamics.^73^ (2) *Similar respiratory physiology*: The physiological parameters, such as tidal volume, respiratory rate, and airway resistance, are similar to those in humans. This similarity allows for more accurate assessments of respiratory function, such as pulmonary function tests (PFTs) and airway resistance measurements, making them more aligned with clinical practices.^74^, ^75^ (3) *Capability for complex operations and long‐term observation*: Sufficient airway size permits testing of clinical‐grade intraluminal devices (e.g., varied stent sizes/types, balloons, ablation tools) and endoscopic procedures (bronchoscopic laser, electrocautery, cryotherapy, injectable therapies). This facilitates long‐term monitoring of stenosis progression, stent‐related complications (granulation, migration, fracture, infection), and therapeutic efficacy.^76^, ^77^, ^78^, ^79^, ^80^

**Limitations:** Limitations include high feeding costs, requirement for specialized facilities, and operator proficiency in advanced bronchoscopic techniques.^63^, ^64^

Therefore, large animals such as pigs and dogs can be used for: (1) *preclinical device evaluation*: These animals can help assess the safety and effectiveness of endoluminal therapeutic devices, including stents, balloon‐expandable catheters, and ablation devices. This involves evaluating the feasibility of device insertion and removal, radial support, adherence to the wall, risk of migration, biocompatibility, and the impact on airway function, as well as the ability to dilate or inhibit stenosis. (2) *Advanced preclinical studies*: Large animals can be used to explore the feasibility of complex therapeutic regimens, such as stents combined with drug coatings or local drug injections, as well as sequential treatment strategies. They also allow researchers to study the biological response to long‐term implantation, such as epithelialization and the mechanisms of restenosis. (3) *Surgical technique training and validation*: These animals provide a valuable platform for doctors to simulate clinical environments for operational training and to validate the feasibility and safety of new surgical techniques.

### 4.3 Featured supplementary model: Rabbit

Rabbits serve as an excellent experimental model for tracheal research due to their anatomical and functional similarities to humans, positioned midway between rodents and larger animals in terms of size and structural complexity. Compared to pigs and dogs, they offer practical advantages, including lower acquisition costs and reduced maintenance expenses, while providing superior tracheal maneuverability over rodent models. Overall, rabbits offer a beneficial balance between the relevance and utility of tracheal research and the feasibility and cost of handling.^37^, ^68^, ^81^, ^82^, ^83^ Therefore, they are an ideal and commonly used option when studies require a larger, more maneuverable, and clinically relevant trachea than that of rodent models, particularly when budgetary, facility, or ethical considerations limit the use of larger animals (Table 5).

**TABLE 5:** Comparative advantages, limitations, and applications of major benign airway stenosis animal models.

Species	Advantages	Limitations	Application scenarios	Modeling duration	References
Mouse/rat	Low cost Transgenic availability Rich immunological tools	Anatomically dissimilar to humans Accelerated disease kinetics Not suitable for device testing	Mechanistic studies, early‐stage drug screening	7–21 days	[1, 26, 27, 28, 29, 30, 31, 32, 50, 62, 63, 64, 65, 66, 67, 68, 69, 70, 71, 72]
Pig/canine	High anatomical/physiological fidelity Suitable for clinical‐grade device testing and complex procedures	Very high cost, requires specialized facilities and surgical expertise	Preclinical device evaluation (stents, balloons), advanced therapeutic regimens, surgical training	12–21 days	[33, 34, 35, 36, 63, 64, 73, 74, 75, 76, 77, 78, 79, 80]
Rabbit	Good balance of anatomical relevance, cost, and tracheal size/maneuverability	Not as physiologically similar as large animals, limited genetic tools	Establishing basic injury‐response relationships, mid‐throughput therapeutic evaluation	7 days–6 weeks	[37, 38, 39, 40, 41, 42, 43, 44, 45, 46, 47, 48, 51, 68, 81, 82, 83]
Ferret	The airway size is similar to that of neonates	Less common model, limited commercial reagents and historical data	Used for the research on BAS in neonates	3 days	[49]

Abbreviation: BAS, benign airway stenosis.

## 5 GAPS OF AVAILABLE MODELS AND FUTURE PERSPECTIVE CHALLENGES

Although more animals and methods are currently used for BAS modeling, still many challenges need to be addressed and standardized for future development.

### 5.1 Gaps of available animal models

*Differences between existing animal models and actual clinical conditions*: (1) Most models rely on acute mechanical/chemical injuries (e.g., brushing, electrocautery, chemical burns), with evaluations conducted within days to weeks (7–28 days) post‐procedure, primarily capturing early‐stage stenosis development. However, clinically significant scarring typically occurs around 6–8 weeks after the initial injury.^6^, ^22^ Consequently, current animal models fail to reliably replicate human chronic bronchial anastomotic stenosis. This clinical condition can persist for months or years. They also fail to reproduce the restenosis seen after interventions such as balloon dilation or laser ablation. (2) Patients with BAS caused by mechanical ventilation often have multiple underlying diseases.^84^ Existing animal models do not adequately account for the individual differences in patients, such as their various underlying diseases and comorbidities. (3) There is a lack of models that can simulate specific etiologies of BAS, such as recurrent polychondritis or Wegener's granulomatosis.^85^

Problems faced in model standardization and normalization: (1) *Injury parameter variability*: The tools, force, duration, and site of injury used by different laboratories vary significantly. This inconsistency makes it challenging to compare and reproduce results. (2) *Assessment inconsistencies*: There are several methods to assess stenosis, such as visual endoscopic grading, histological measurements, imaging reconstruction, and lung function tests. However, there is no consensus on best practices or quantitative criteria for these assessments.^26^, ^49^

### 5.2 Developmental strategies for the next generation of BAS animal models

(1) *Etiology‐specific model development*: Explore models that are immune‐mediated (e.g., induced autoimmunity), postinfectious (e.g., chronic inflammation induced by specific pathogens), or incorporate the underlying disease (e.g., diabetes mellitus, gastroesophageal reflux). (2) *Injury method optimization*: Design milder and more controllable chronic injury regimens that mimic the course of long‐term clinical irritation. (3) *Prolonged observation periods*: Establish chronic stenosis models that can be maintained consistently for months or longer, allowing for the study of long‐term remodeling and therapeutic effects. (4) *Standardized protocol establishment*: Encourage the development and widespread adoption of standardized injury methods and systematic assessment protocols for various models (e.g., rat tracheal brush scraping, porcine stenting postimplantation for sarcoidosis). (5) *Objective quantification tools*: Promote the use of micro‐CT, OCT, etc., to perform accurate three‐dimensional reconstruction of the airway and stenosis calculation and utilize automated image analysis for histological quantification, including measurements like percentage collagen area, inflammatory cell counts, and epithelial thickness. (6) *Shared resource infrastructure*: Facilitate the sharing of model data and biological samples among different research institutions to validate and compare results effectively.

In conclusion, the diverse modeling methods and evaluation approaches used in existing BAS animal models offer research flexibility across various experimental needs. However, limitations persist in modeling standardization and assessment consistency. Future investigations should prioritize the development of modeling strategies that are more aligned with clinical pathology. Additionally, we should improve the dynamic monitoring and quantification standards for assessment indices and enhance the accuracy and predictive value of these models by integrating emerging technologies, such as multiomics analysis. These advancements will lay a solid foundation for understanding the pathogenesis of BAS and developing new diagnostic and therapeutic technologies, ultimately driving substantive breakthroughs in clinical management.

## AUTHOR CONTRIBUTIONS

**Wusheng Zhang:** Conceptualization; writing – original draft. **Yilin Chen:** Investigation; visualization. **Chengcheng Yang:** Conceptualization; visualization. **Yuchao Dong:** Supervision. **Haidong Huang:** Supervision. **Hui Shi:** Funding acquisition; supervision. **Chong Bai:** Conceptualization; funding acquisition; project administration; supervision.

## FUNDING INFORMATION

This work was supported by the National Natural Science Foundation of China with grant numbers 82270112 (Chong Bai) and 82000102 (Hui Shi).

## CONFLICT OF INTEREST STATEMENT

The authors declare no competing or financial interests.

## ETHICS APPROVAL AND CONSENT TO PARTICIPATE

As this is a review article, ethics approval and consent to participate are not applicable.

## CONSENT FOR PUBLICATION

All authors have given their consent for the publication of this manuscript.

## References

[1] Wei JM , Chen Y , Feng TM , et al. miR‐34c‐5p inhibited fibroblast proliferation, differentiation and epithelial‐mesenchymal transition in benign airway stenosis via MDMX/p53 pathway. Funct Integr Genomics. 2024;24(2):37. 38374244 10.1007/s10142-024-01317-y PMC10876495

[2] Yang ZY , Zhou XL , Pan WY , Zeng DX , Jiang JH . Clinical analysis of 32 cases of subglottic benign airway stenosis treated with montgomery T silicone stent. Can Respir J. 2024;2024(1):2145560. 39444845 10.1155/2024/2145560 PMC11498979

[3] Huang ZJ , Wei P , Gan LM , et al. Expression of histone deacetylase 2 in tracheal stenosis models and its relationship with tracheal granulation tissue proliferation. Exp Ther Med. 2021;21(5):444. 33747180 10.3892/etm.2021.9872 PMC7967890

[4] Yang MY , Li H , Zhou YZ , Li H , Wei HF , Cheng QH . Airway collapse hinders recovery in bronchoscopy therapy for postintubation tracheal stenosis patients. EurArchOtorhinolaryngol. 2024;281(6):3061‐3069. 10.1007/s00405-024-08602-3 PMC11065913 38582815

[5] Pappal RB , Burruss CP , Witt MA , et al. Risk factors for developing subglottic and tracheal stenosis from the medical intensive care unit. Laryngoscope Investig Otolaryngol. 2023;8(3):699‐707. 10.1002/lio2.1051 PMC10278098 37342110

[6] Qiu XJ , Zhang J , Wang T , Pei YH , Xu M . Nonstent combination interventional therapy for treatment of benign cicatricial airway stenosis. Chin Med J. 2015;128(16):2154‐2161. 26265607 10.4103/0366-6999.162496 PMC4717983

[7] Kumar A , Asaf BB , Puri HV , Abdellateef A . Resection and anastomosis for benign tracheal stenosis: Single institution experience of 18 cases. Lung India. 2017;34(5):420‐426. 28869225 10.4103/0970-2113.213834 PMC5592752

[8] Chen DF , Chen Y , Zhong CH , Chen XB , Li SY . Long‐term efficacy and safety of the Dumon stent for benign tracheal stenosis: a meta‐analysis. J Thorac Dis. 2021;13(1):82‐91. 33569188 10.21037/jtd-20-2327 PMC7867818

[9] Lian TM , Liang C , Yu SY , et al. Bronchial balloon dilatation combined with cryotherapy for tuberculous cicatricial central airway stenosis, with Adobe Photoshop for the degree measurement: a multicenter, retrospective study. Heliyon. 2023;9(11):e22326. 38045188 10.1016/j.heliyon.2023.e22326 PMC10689942

[10] Sachs N , Papaspyropoulos A , van Zomer‐ Ommen DD , et al. Long‐term expanding human airway organoids for disease modeling. EMBO J. 2019;38(4):e100300. 30643021 10.15252/embj.2018100300 PMC6376275

[11] Vazquez‐Armendariz AI , Tata PR . Recent advances in lung organoid development and applications in disease modeling. J Clin Invest. 2023;133(22):e170500. 37966116 10.1172/JCI170500 PMC10645385

[12] Jiang SP , Lin BQ , Zhou XQ , et al. Airway organoid models as pivotal tools for unraveling molecular mechanisms and therapeutic targets in respiratory diseases: a literature review. Ther Clin Risk Manag. 2025;21:975‐986. 40584024 10.2147/TCRM.S526727 PMC12205757

[13] Matkovic Leko I , Schneider RT , Thimraj TA , et al. A distal lung organoid model to study interstitial lung disease, viral infection and human lung development. Nat Protoc. 2023;18(7):2283‐2312. 37165073 10.1038/s41596-023-00827-6 PMC11486529

[14] Srivastava SK , Foo GW , Aggarwal N , Chang MW . Organ‐on‐chip technology: opportunities and challenges. Biotechnol Notes. 2024;5:8‐12. 39416695 10.1016/j.biotno.2024.01.001 PMC11446384

[15] Artzy Schnirman A , Hobi N , Schneider‐Daum N , Guenat OT , Lehr C‐M , Sznitman J . Advanced in vitro lung‐on‐chip platforms for inhalation assays: from prospect to pipeline. Eur J Pharm Biopharm. 2019;144:11‐17. 31499161 10.1016/j.ejpb.2019.09.006 PMC7611793

[16] Koceva H , Amiratashani M , Akbarimoghaddam P , et al. Deciphering respiratory viral infections by harnessing organ‐on‐chip technology to explore the gut–lung axis. Open Biol. 2025;15(3):240231. 40037530 10.1098/rsob.240231 PMC11879621

[17] Dichtl S , Posch W , Wilflingseder D . The breathtaking world of human respiratory in vitro models: investigating lung diseases and infections in 3D models, organoids, and lung‐on‐chip. Eur J Immunol. 2024;54(3):e2250356. 38361030 10.1002/eji.202250356

[18] Fisher CR , Mba Medie F , Luu RJ , et al. A high‐throughput, high‐containment human primary epithelial airway organ‐on‐Chip platform for SARS‐CoV‐2 therapeutic screening. Cells. 2023;12(22):2639. 37998374 10.3390/cells12222639 PMC10669988

[19] Auletta B , Chiolerio P , Cecconi G , et al. Tissue‐engineered neuromuscular organoids. Commun Biol. 2025;8(1):1074. 40684029 10.1038/s42003-025-08484-z PMC12276242

[20] Norouzi S , Saveh Shemshaki N , Norouzi E , et al. Recent advances in biomaterials for tissue‐engineered constructs: essential factors and engineering techniques. Mater Today Chem. 2024;37:102016.

[21] Davis RJ , Hillel AT . Inflammatory pathways in the pathogenesis of iatrogenic laryngotracheal stenosis: what do we know? Transl Cancer Res. 2020;9(3):2108‐2116. 35117566 10.21037/tcr.2020.01.21 PMC8797408

[22] Carpenter DJ , Hamdi OA , Finberg AM , Daniero JJ . Laryngotracheal stenosis: mechanistic review. Head Neck. 2022;44(8):1948‐1960. 35488503 10.1002/hed.27079 PMC9543412

[23] Horejs C . Organ chips, organoids and the animal testing conundrum. Nat Rev Mater. 2021;6(5):1‐2. 10.1038/s41578-021-00313-z PMC8072732 33936776

[24] Françoise BS , Montagutelli X . Animal models are essential to biological research: issues and perspectives. Future Sci OA. 2015;1(4):FSO63. 28031915 10.4155/fso.15.63 PMC5137861

[25] Swearengen JR . Choosing the right animal model for infectious disease research. Animal Models Exp Med. 2018;1(2):100‐108. 10.1002/ame2.12020 PMC6388060 30891554

[26] Khalmuratova R , Kim YS , Kim DW , Shin HW . A differentiated epithelial layer graft improves fibrosis and survival in airway stenosis via IL‐37. Allergy. 2024;80(5):1468‐1472. 39711088 10.1111/all.16452 PMC12105059

[27] Chen YL , Yang C , Miao YS , et al. Macrophage STING signaling promotes fibrosis in benign airway stenosis via an IL6‐STAT3 pathway. Nat Commun. 2025;16(1):289. 39753529 10.1038/s41467-024-55170-5 PMC11698984

[28] Fan YH , Li X , Fang X , et al. Antifibrotic role of Nintedanib in tracheal stenosis after a tracheal wound. Laryngoscope. 2021;131(9):E2496‐E2505. 34000066 10.1002/lary.29618

[29] He CY , Li AM , Gu L , et al. Repression of Smad3 by silencing Robo1 attenuates epithelial‐mesenchymal transition in tracheobronchial stenosis. Chin Med J. 2023;136(17):2125‐2127. 37455334 10.1097/CM9.0000000000002528 PMC10476802

[30] Liao JX , Gan YL , Peng MY , et al. GDF15 alleviates the progression of benign tracheobronchial stenosis by inhibiting epithelial‐mesenchymal transition and inactivating fibroblasts. Exp Cell Res. 2022;421(2):113410. 36336027 10.1016/j.yexcr.2022.113410

[31] Chen S , Zhang W , Ning YY , Dong YC , Li Q , Bai C . Establishment of a benign tracheal stenosis model in rats by nylon brush scraping induced mechanical injury. Chin J Respir Crit Care Med. 2019;18(3):265‐270.

[32] Mizokami D , Araki K , Tanaka N , et al. Tacrolimus prevents laryngotracheal stenosis in an acute‐injury rat model. Laryngoscope. 2015;125(6):E210‐E215. 25647147 10.1002/lary.25178

[33] Kim JH , Ahn JJ , Jegal Y , et al. Rapid establishment of tracheal stenosis in pigs using endotracheal tube cuff overpressure and electrocautery. Curr Med Sci. 2021;41(2):329‐335. 33877550 10.1007/s11596-021-2351-0

[34] Zhou ZQ , Su ZQ , Sun W , et al. Postintubation tracheal stenosis evaluated by endobronchial optical coherence tomography: a canine model study. Respiration. 2020;99(6):500‐507. 32485723 10.1159/000506882

[35] Li F , Li PP , Cai ZG , et al. Establishment of two canine models of benign airway stenosis and the effect of mitomycin C on airway stenosis. Int J Pediatr Otorhinolaryngol. 2022;159:111205. 35700689 10.1016/j.ijporl.2022.111205

[36] Chen XB , Wang WH , Ye YS , et al. The wound healing of autologous regenerative factor on recurrent benign airway stenosis: a canine experimental and pilot study. Respiration. 2024;103(3):111‐123. 38342097 10.1159/000536007

[37] Zhang GY , Wang JM , Zeng YM . A modified rabbit model of tracheal stenosis and a household endoscope. More simplicity and accessibility. Acta Cir Bras. 2020;35(11):e351104. 33331454 10.1590/ACB351104 PMC7748079

[38] Wistermayer P , Escalante D , McIlwain W , Rogers DJ . A randomized controlled trial of dexamethasone as a prophylactic treatment for subglottic stenosis in a rabbit model. Ann Otol Rhinol Laryngol. 2020;130(2):3489420946773. 10.1177/0003489420946773 32749146

[39] Wang LH , Zhang J , Chen N , Zhang YY , Xu M , Yue YM . The pilot study of the effect of paclitaxel by local application on scar formation after airway injury inrabbits. Zhonghua Jie He He Hu Xi Za Zhi. 2013;36(3):202‐206. 23856144

[40] Qin EY , Xu MP , Gan LM , et al. Erythromycin combined with corticosteroid reduced inflammation and modified trauma‐induced tracheal stenosis in a rabbit model. Ther Adv Respir Dis. 2018;12:1753466618773707. 29781361 10.1177/1753466618773707 PMC5966843

[41] Wilson J , Utz E , Marvin K , Schwartz I , Johnson C , Gaudreau P . Rabbit model of consistently survivable subglottic stenosis using a modified brush technique. Int J Pediatr Otorhinolaryngol. 2020;139:110474. 33130465 10.1016/j.ijporl.2020.110474

[42] Zhang J , Liu YH , Yang ZY , et al. The role of tracheal wall injury in the development of benign airway stenosis in rabbits. Sci Rep. 2023;13(1):3144. 36823432 10.1038/s41598-023-29483-2 PMC9950474

[43] Lin H , Ainiwaer M , Jiang Z , Wang Z , Liu J , Chen F . Comparative evaluation of mechanical injury methods for establishing stable tracheal stenosis animal models. Sci Rep. 2024;14(1):2383. 38287058 10.1038/s41598-024-52230-0 PMC10824766

[44] McIlwain WR , Wistermayer PR , Swiss TP , Marko ST , Ieronimakis NM , Rogers DJ . Reproducing severe acute subglottic stenosis in a rabbit model. Int J Pediatr Otorhinolaryngol. 2017;103:142‐146. 29224757 10.1016/j.ijporl.2017.10.011

[45] Lee JY , Son SJ , Choi SH , Cho DW . The healing effect of platelet‐rich plasma (PRP) jelly in rabbits undergoing tracheal resection and anastomosis. In vivo (Athens, Greece). 2019;33(1):75‐78. 30587605 10.21873/invivo.11441 PMC6364083

[46] Aydogmus U , Topkara A , Akbulut M , et al. Effectiveness of palatal mucosa graft in surgical treatment of sub‐glottic stenosis. Clin Exp Otorhinolaryngol. 2016;9(4):358‐365. 27416739 10.21053/ceo.2015.01508 PMC5115148

[47] Lee HS , Kim SW , Oak C , et al. Rabbit model of tracheal stenosis using cylindrical diffuser. Lasers Surg Med. 2017;49(4):372‐379. 27862085 10.1002/lsm.22615

[48] Schweiger C , Hart CK , Tabangin ME , et al. Development of a survival animal model for subglottic stenosis. Laryngoscope. 2018;129(4):989‐994. 30208212 10.1002/lary.27441

[49] Tubbs KJ , Silva RC , Ramirez HE , Castleman WL , Collins WO . A comparison of two methods of endoscopic dilation of acute subglottic stenosis using a ferret model. Laryngoscope. 2013;123(1):253‐258. 22961260 10.1002/lary.23508

[50] Niu F , Sun B , Yu Y , et al. SPP1‐mediated crosstalk between macrophage and fibroblasts promotes benign airway stenosis. Arch Biochem Biophys. 2025;774:110627. 41015147 10.1016/j.abb.2025.110627

[51] Luo L , Huang J , Chen D , et al. Airway basal stem cell‐derived extracellular vesicles drive ECM remodeling and suppress fibroblasts activation via the miR‐30a‐5p/FAP axis in benign tracheal stenosis. J Adv Res. 2025. doi:10.1016/j.jare.2025.08.014 40812587

[52] Huang CH , Lichtarge S , Fernandez D . Integrative whole slide image and spatial transcriptomics analysis with QuST and QuPath. NPJ Precis Oncol. 2025;9(1):70. 40075141 10.1038/s41698-025-00841-9 PMC11904241

[53] Bankhead P , Loughrey MB , Fernández JA , et al. QuPath: open source software for digital pathology image analysis. Sci Rep. 2017;7(1):16878. 29203879 10.1038/s41598-017-17204-5 PMC5715110

[54] Beeche C , Singh JP , Leader JK , et al. Super U‐net: a modularized generalizable architecture. Pattern Recogn. 2022;128:108669. 10.1016/j.patcog.2022.108669 PMC9070860 35528144

[55] Azad R , Aghdam EK , Rauland A , et al. Medical image segmentation review: the success of U‐net. IEEE Trans Pattern Anal Mach Intell. 2024;46(12):10076‐10095. 39167505 10.1109/TPAMI.2024.3435571

[56] Mohamady YK , Geudens V , De Fays C , et al. Computational fluid dynamics of small airway disease in chronic obstructive pulmonary disease. EBioMedicine. 2025;114:105670. 40174553 10.1016/j.ebiom.2025.105670 PMC11999283

[57] Schwab AD , Wyatt TA , Schanze OW , et al. Lung‐delivered IL‐10 mitigates Lung inflammation induced by repeated endotoxin exposures in male mice. Physiol Rep. 2025;13(4):e70253. 39980189 10.14814/phy2.70253 PMC11842461

[58] Vermaut A , Aerts G , Willems L , et al. Pulmonary arteriole narrowing in end‐stage cystic fibrosis lungs occurs with and without small airway disease. J Cyst Fibros. 2025;24(5):946‐953. 40340199 10.1016/j.jcf.2025.04.002

[59] Daneshpour Moghadam S , Maris B , Mokhtari A , Daffara C , Fiorini P . OCT in oncology and precision medicine: from nanoparticles to advanced technologies and AI. Bioengineering. 2025;12(6):650. 40564467 10.3390/bioengineering12060650 PMC12189282

[60] Xu Y , Soundararajan S , Randell SH , et al. In vivo assessment of airway wall compliance during inhalation injury response using anatomical optical coherence elastography. J Biomed Opt. 2025;30(7):076001. 40621167 10.1117/1.JBO.30.7.076001 PMC12223793

[61] Hou P , Chen H , Liang S , et al. Targeting fibroblast activation protein for molecular imaging of fibrotic remodeling in pulmonary arterial hypertension. J Nucl Med. 2025;66(1):98‐103. 39753365 10.2967/jnumed.124.268376

[62] Loiseau A , Marcoux GR , Maranda C , Bertrand N , Boisselier E . Animal models in eye research: focus on corneal pathologies. Int J Mol Sci. 2023;24(23):16661. 38068983 10.3390/ijms242316661 PMC10706114

[63] Milani Nejad N , Janssen PML . Small and large animal models in cardiac contraction research: advantages and disadvantages. PharmacolTher. 2014;141(3):235‐249. 10.1016/j.pharmthera.2013.10.007 PMC3947198 24140081

[64] Gonzalez LM , Moeser AJ , Blikslager AT . Porcine models of digestive disease: the future of large animal translational research. Transl Res. 2015;166(1):12‐27. 25655839 10.1016/j.trsl.2015.01.004 PMC4458388

[65] Cao Y , Chen QQ , Liu YN , Jin LB , Peng RY . Research progress on the construction and application of a diabetic zebrafish model. Int J Mol Sci. 2023;24(6):5195. 36982274 10.3390/ijms24065195 PMC10048833

[66] Kheirallah AK , Miller S , Hall IP , Sayers I . Translating Lung Function Genome‐Wide Association Study (GWAS) findings: new insights for lung biology. Adv Genet. 2016;93:57‐145. 26915270 10.1016/bs.adgen.2015.12.002

[67] Eleonore F . Animals in respiratory research. Int J Mol Sci. 2024;25(5):2903. 38474149 10.3390/ijms25052903 PMC10931704

[68] Tanner L , Single AB . Animal models reflecting chronic obstructive pulmonary disease and related respiratory disorders: translating pre‐clinical data into clinical relevance. J Innate Immun. 2020;12(3):203‐225. 31527372 10.1159/000502489 PMC7265725

[69] Ribitsch I , Baptista PM , LangeConsiglio A , et al. Large animal models in regenerative medicine and tissue engineering: to do or not to do. Front Bioeng Biotechnol. 2020;8:972. 32903631 10.3389/fbioe.2020.00972 PMC7438731

[70] Fehrenbach H , Wagner C , Wegmann M . Airway remodeling in asthma: what really matters. Cell Tissue Res. 2017;367(3):551‐569. 28190087 10.1007/s00441-016-2566-8 PMC5320023

[71] Alessandrini F , Musiol S , Schneider E , Blanco Pérez F , Albrecht M . Mimicking antigen‐driven asthma in rodent models‐how close can we get? Front Immunol. 2020;11:575936. 33101301 10.3389/fimmu.2020.575936 PMC7555606

[72] Rydell‐Törmänen K , Johnson JR . The applicability of mouse models to the study of human disease. Methods Mol Biol (Clifton, NJ). 2019;1940:3‐22. 10.1007/978-1-4939-9086-3_1 PMC7121329 30788814

[73] Huang Z , Wang L , Zhang CX , et al. Biomechanical strength dependence on mammalian airway length. J Thorac Dis. 2021;13(2):918‐926. 33717564 10.21037/jtd-20-2970 PMC7947550

[74] Pulido L , Burgos D , García Morato J , Luna CM . Does animal model on ventilator‐associated pneumonia reflect physiopathology of sepsis mechanisms in humans? Ann Transl Med. 2017;5(22):452. 29264369 10.21037/atm.2017.11.35 PMC5721223

[75] Kulkarni HS , Lee JS , Bastarache JA , et al. Update on the Features and Measurements of Experimental Acute Lung Injury in Animals: An Official American Thoracic Society Workshop Report. Am J Respir Cell Mol Biol. 2022;66(2):e1‐e14. 35103557 10.1165/rcmb.2021-0531ST PMC8845128

[76] Lukas M , Kolar M , Ryska O , et al. A novel postgraduate endoscopic course using a large animal model of secondary Crohn's disease stricture. Surg Endosc. 2021;35(6):1‐6. 33661380 10.1007/s00464-021-08360-x

[77] Chen R , Pye JS , Li J , Little CB , Li JJ . Multiphasic scaffolds for the repair of osteochondral defects: outcomes of preclinical studies. Bioact Mater. 2023;27:505‐545. 37180643 10.1016/j.bioactmat.2023.04.016 PMC10173014

[78] Zong JB , He QW , Liu YX , Qiu M , Wu JH , Hu B . Advances in the development of biodegradable coronary stents: a translational perspective. Mater Today Bio. 2022;16:100368. 10.1016/j.mtbio.2022.100368 PMC9352968 35937578

[79] Maynard LH , Humbert O , Peterson CW , Kiem H . Genome editing in large animal models. Mol Ther. 2021;29(11):3140‐3152. 34601132 10.1016/j.ymthe.2021.09.026 PMC8571486

[80] Hiratsuka T , Inomata M . A novel animal model of colonic stenosis to aid the development of new stents for colon strictures. Surg Endosc. 2021;36(5):1‐8. 34159466 10.1007/s00464-021-08618-4

[81] Han MN , Kim JH , Choi SH . Evaluation of biomechanical properties and morphometric structures of the trachea in pigs and rabbits. In vivo (Athens, Greece). 2022;36(4):1718‐1725. 35738586 10.21873/invivo.12884 PMC9301417

[82] Kim JH , Choi JY , Yoon HY . A rabbit model of tracheal collapse for optimal self‐expanding metal stents. J Vet Med Sci. 2023;85(3):386‐392. 36740259 10.1292/jvms.22-0167 PMC10076205

[83] Kamaruzaman NA , Kardia E , Kamaldin NA , Latahir AZ , Yahaya BH . The rabbit as a model for studying lung disease and stem cell therapy. Biomed Res Int. 2013;2013:691830. 23653896 10.1155/2013/691830 PMC3638694

[84] Gelbard A , Francis DO , Sandulache VC , Simmons JC , Donovan DT , Ongkasuwan J . Causes and consequences of adult laryngotracheal stenosis. Laryngoscope. 2015;125(5):1137‐1143. 25290987 10.1002/lary.24956 PMC4562418

[85] Ravikumar N , Ho E , Wagh A , Murgu S . The role of bronchoscopy in the multidisciplinary approach to benign tracheal stenosis. J Thorac Dis. 2023;15(7):3998‐4015. 37559626 10.21037/jtd-22-1734 PMC10407490

